# Germline Polymorphisms and Length of Survival of Nasopharyngeal Carcinoma: An Exome‐Wide Association Study in Multiple Cohorts

**DOI:** 10.1002/advs.201903727

**Published:** 2020-03-20

**Authors:** Yun‐Miao Guo, Jie‐Rong Chen, Yan‐Chun Feng, Melvin L. K. Chua, Yanni Zeng, Edwin Pun Hui, Allen K. C. Chan, Lin‐Quan Tang, Lin Wang, Qian Cui, Hui‐Qiong Han, Chun‐Ling Luo, Guo‐Wang Lin, Yan Liang, Yang Liu, Zhong‐Lian He, Yu‐Xiang Liu, Pan‐Pan Wei, Chu‐Jun Liu, Wan Peng, Bo‐Wei Han, Xiao‐Yu Zuo, Enya H. W. Ong, Eugenia L. L. Yeo, Kar Perng Low, Gek San Tan, Tony K. H. Lim, Jacqueline S. G. Hwang, Bo Li, Qi‐Sheng Feng, Xiaojun Xia, Yun‐Fei Xia, Josephine Ko, Wei Dai, Maria L. Lung, Anthony T. C. Chan, Dennis Y. M. Lo, Mu‐Sheng Zeng, Hai‐Qiang Mai, Jianjun Liu, Yi‐Xin Zeng, Jin‐Xin Bei

**Affiliations:** ^1^ Sun Yat‐sen University Cancer Center State Key Laboratory of Oncology in South China Collaborative Innovation Center for Cancer Medicine Guangdong Key Laboratory of Nasopharyngeal Carcinoma Diagnosis and Therapy Guangzhou 510060 P. R. China; ^2^ Department of Pathology The Affiliated Hospital of Guangdong Medical University Zhanjiang 524001 P. R. China; ^3^ Guangdong Provincial People's Hospital Guangdong Academy of Medical Sciences Guangzhou 510080 P. R. China; ^4^ Division of Radiation Oncology and Division of Medical Sciences National Cancer Centre Singapore Singapore 169610 Singapore; ^5^ Duke‐NUS Medical School Singapore 169857 Singapore; ^6^ Faculty of Forensic Medicine Guangdong Province Translational Forensic Medicine Engineering Technology Research Centre Guangdong Province Key Laboratory of Brain Function and Disease Zhongshan School of Medicine Sun Yat‐sen University Guangzhou 510080 P. R. China; ^7^ Department of Clinical Oncology State Key Laboratory of Translational Oncology Prince of Wales Hospital The Chinese University of Hong Kong Shatin, New Territories Hong Kong SAR P. R. China; ^8^ Department of Chemical Pathology Li Ka Shing Institute of Health Sciences State Key Laboratory of Translational Oncology The Chinese University of Hong Kong Shatin, New Territories Hong Kong SAR P. R. China; ^9^ Department of Oncology The First Affiliated Hospital of Zhengzhou University Zhengzhou 450052 P. R. China; ^10^ Translational Pathology Centre Department of Molecular Pathology Singapore General Hospital Singapore 169856 Singapore; ^11^ Department of Anatomical Pathology Singapore General Hospital Singapore 169856 Singapore; ^12^ Department of Biochemistry and Molecular Biology Zhongshan School of Medicine Sun Yat‐sen University Guangzhou 510080 P. R. China; ^13^ RNA Biomedical Institute Sun Yat‐sen Memorial Hospital Sun Yat‐sen University Guangzhou 510120 P. R. China; ^14^ Department of Clinical Oncology University of Hong Kong Pok Fu Lam Hong Kong SAR P. R. China; ^15^ Genome Institute of Singapore Genome Building Singapore 138672 Singapore; ^16^ Center for Precision Medicine Sun Yat‐sen University Guangzhou 510080 P. R. China

**Keywords:** biomarkers, cancer prognosis, germline polymorphisms, nasopharyngeal carcinoma, RPA1, single nucleotide polymorphisms

## Abstract

Germline polymorphisms are linked with differential survival outcomes in cancers but are not well studied in nasopharyngeal carcinoma (NPC). Here, a two‐phase association study is conducted to discover germline polymorphisms that are associated with the prognosis of NPC. The discovery phase includes two consecutive hospital cohorts of patients with NPC from Southern China. Exome‐wide genotypes at 246 173 single nucleotide polymorphisms (SNPs) are determined, followed by survival analysis for each SNP under Cox proportional hazard regression model. Candidate SNP is replicated in another two independent cohorts from Southern China and Singapore. Meta‐analysis of all samples (*n* = 5553) confirms that the presence of rs1131636‐T, located in the 3′‐UTR of *RPA1*, confers an inferior overall survival (HR = 1.33, 95% CI = 1.20–1.47, *P* = 6.31 × 10^−8^). Bioinformatics and biological assays show that rs1131636 has regulatory effects on upstream *RPA1*. Functional studies further demonstrate that RPA1 promotes the growth, invasion, migration, and radioresistance of NPC cells. Additionally, miR‐1253 is identified as a suppressor for RPA1 expression, likely through regulation of its binding affinity to rs1131636 locus. Collectively, these findings provide a promising biomarker aiding in stratifying patients with poor survival, as well as a potential drug target for NPC.

## Graphical abstract

This study reveals a functional germline polymorphism at *RPA1* to be associated with survival outcome of patients with nasopharyngeal carcinoma (NPC). The study also shows that RPA1 is able to regulate tumor progression and radioresistance in NPC cells. Together, the study proposes the genetic variation of *RPA1* as a promising germline biomarker for precise treatment planning and prognosis of NPC.

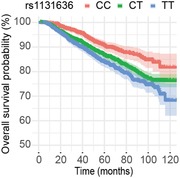

## 1 Introduction

Nasopharyngeal carcinoma (NPC) is an Epstein–Barr virus related malignancy with unique ethnic and geographic distribution, which is prevalent in Southern China, Southeastern Asia, and Northern Africa.^[^ ^1^ ^]^ Radiotherapy is the primary therapeutic modality for NPC because of the radiosensitive nature of its tumor cells and the deep‐seated anatomical position. Survival outcomes in patients with NPC have improved substantially,^[^ ^2^ ^]^ largely due to the widespread implementation of intensity‐modulated radiotherapy (IMRT) and the addition of platinum‐based chemotherapy in patients with loco/regionally advanced disease.^[^ ^3^ ^]^ Nonetheless, current treatment strategy and risk stratification for clinical outcomes remain confined to the conventional tumor‐node‐metastasis (TNM) classification system for NPC.^[^ ^4^ ^]^ Patients with the same clinical stage have different outcomes after receiving similar treatments, indicating heterogeneity among patients with the same clinical stage as categorized by the TNM system and the limitation of the system in predicting the treatment outcomes.^[^ ^5^ ^]^ Disease recurrence and metastasis are major causes of treatment failure and thus poor survival in NPC. Therefore, it is important to identify effective biomarkers or indicators and reveal the underlying mechanisms for precise treatment planning and accurate prognosis of patients with NPC.

Genetic polymorphisms among individuals contribute to their phenotypic differences, such as disease predisposition, treatment response, and survival.^[^ ^6^ ^]^ In NPC, genetic polymorphisms have been associated with its predisposition and development.^[^ ^7^ ^]^ Nonetheless, the exact mechanisms underpinning how genetic polymorphisms drive tumor behavior and eventual clinical outcomes are not well elucidated. Up to date, a few association studies have reported that genetic variants in candidate genes such as *MCP‐1*, *HLA‐G*, *TP53*, *CELF2*, and *CXCL12* are associated with prognosis of NPC.^[^ ^8^ ^]^ However, the robustness of these findings remains limited by the small study sample sizes and the restriction of a candidate gene approach.

Here, to investigate germline polymorphisms that might contribute to NPC prognosis, we conducted a large exome‐wide association study of 31 870 common single nucleotide polymorphisms (SNPs) with survival in two consecutive cohorts from Southern China involving 3257 patients with NPC and observed an association of a germline polymorphism in *RPA1* gene with survival. The association was replicated in two additional cohorts involving 2296 patients with NPC recruited from Southern China and Singapore. We further demonstrated the functional relevance of RPA1 on tumor aggression and radioresistance as well as the mechanism of the germline SNP involved in regulating RPA1 expression.

## 2 Results

### 2.1 Patient Characteristics and Survival Status

Clinical characteristics of patients with NPC were summarized for all sample collections (**Table** **1**; Table S1, Supporting Information). At the time of last follow‐up, we recorded a total of 777 (14.0%) deaths. Median follow‐up duration of patients ranged from 48.8 to 94.5 months for the cohorts. We observed superior overall survival in patients who underwent IMRT (hazard ratio or HR = 0.49, 95% confidence interval or CI = 0.42–0.57, *P* < 0.0001) and concurrent chemoradiotherapy (CCRT; HR = 0.76, 95% CI = 0.66–0.88, *P* = 0.0002), as compared with those did not (Table 1), which is consistent with the previous findings that utilization of IMRT or CCRT led to superior survival in patients with NPC.^[^ ^3a,b^ ^]^

**Table 1:** Clinical characteristics and overall survival of patients with NPC

Characteristics	Discovery cohorts		Replication cohorts		Combined samples	HR (95% CI)	*P*
	SYSUCC‐1	SYSUCC‐2	SYSUCC‐3	NCCS			
Total	1471	1786	1751	545	5553		
Death	346 (23.5%)	256 (14.3%)	106 (6.1%)	69 (12.7%)	777 (14%)		
Distant metastasis	197 (13.4%)	186 (10.4%)	161 (9.2%)	72 (13.2%)	616 (11.1%)		
Locoregional relapse	143 (9.7%)	95 (5.3%)	58 (3.3%)	71 (13%)	367 (6.6%)		
Duration	2003.3–2007.12	2008.1–2012.4	2008.4–2015.6	2008.1–2018.6	2003.3–2018.6		
MST, months (IQR)	94.5 (61.6–102)	48.8 (40.6–58)	53.6 (44.9–62.8)	64.4 (29.5–94.8)	55.6 (43.2–73.5)		
Gender							
Male	1074 (73%)	1345 (75.3%)	1278 (73%)	413 (75.8%)	4110 (74%)	0.60 (0.50–0.72)	<0.0001
Female	397 (27%)	441 (24.7%)	473 (27%)	132 (24.2%)	1443 (26%)		
Age (mean ± SD, years)	52.3 ± 11.2	48 ± 11.7	47.2 ± 11.8	51.2 ± 10.9	49.2 ± 11.7	1.82 (1.58–2.11)	<0.0001
Tumor classification							
T1	198 (13.5%)	170 (9.5%)	117 (6.7%)	162 (29.7%)	647 (11.7%)		
T2	295 (20.1%)	333 (18.6%)	306 (17.5%)	126 (23.1%)	1060 (19.1%)	1.45 (1.33–1.57)	<0.0001
T3	700 (47.6%)	871 (48.8%)	924 (52.8%)	156 (28.6%)	2651 (47.7%)		
T4	278 (18.9%)	412 (23.1%)	404 (23.1%)	101 (18.5%)	1195 (21.5%)		
Lymph node metastasis							
N0	360 (24.5%)	233 (13%)	228 (13%)	72 (13.2%)	893 (16.1%)		
N1	446 (30.3%)	725 (40.6%)	702 (40.1%)	182 (33.4%)	2055 (37%)	1.54 (1.41–1.67)	<0.0001
N2	579 (39.4%)	637 (35.7%)	629 (35.9%)	214 (39.3%)	2059 (37.1%)		
N3	86 (5.8%)	191 (10.7%)	192 (11%)	77 (14.1%)	546 (9.8%)		
Distant metastasis							
M0	1441 (98%)	1684 (94.3%)	1712 (97.8%)	540 (99.1%)	5377 (96.8%)	6.13 (4.84–7.75)	<0.0001
M1	30 (2%)	102 (5.7%)	39 (2.2%)	5 (0.9%)	176 (3.2%)		
Clinical stage							
I	80 (5.4%)	59 (3.3%)	44 (2.5%)	41 (7.5%)	224 (4%)		
II	222 (15.1%)	222 (12.4%)	189 (10.8%)	116 (21.3%)	749 (13.5%)		
III	803 (54.6%)	899 (50.3%)	943 (53.9%)	225 (41.3%)	2870 (51.7%)	1.75 (1.64–1.87)	<0.0001
IVA	258 (17.5%)	352 (19.7%)	354 (20.2%)	83 (15.2%)	1047 (18.9%)		
IVB	78 (5.3%)	152 (8.5%)	182 (10.4%)	75 (13.8%)	487 (8.8%)		
IVC	30 (2%)	102 (5.7%)	39 (2.2%)	5 (0.9%)	176 (3.2%)		
IMRT							
No	1234 (83.9%)	747 (41.8%)	0 (0%)	0 (0%)	1981 (35.7%)	0.49 (0.42–0.57)	<0.0001
Yes	237 (16.1%)	1039 (58.2%)	1751 (100%)	545 (100%)	3572 (64.3%)		
CCRT							
No	903 (61.4%)	554 (31%)	261 (14.9%)	156 (28.6%)	1874 (33.7%)	0.76 (0.66–0.88)	0.0002
Yes	568 (38.6%)	1232 (69%)	1490 (85.1%)	389 (71.4%)	3679 (66.3%)		

MST, median survival time; IMRT, intensity modulated radiation therapy; CCRT, concurrent chemoradiotherapy; ICT, induction chemotherapy; ACT, adjuvant chemotherapy. HR and *P* values were derived from univariate Cox proportional hazards regression analyses.

### 2.2 Genetic Variants Associated with NPC Prognosis

**Figure 1:**
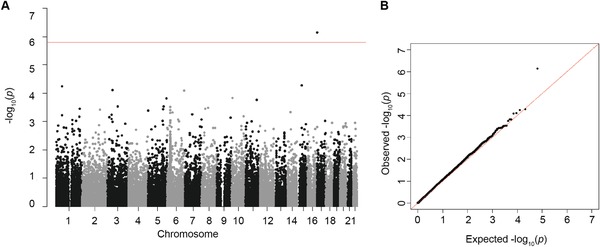
Results of meta‐analysis for 31 870 autosomal SNPs and overall survival time of patients with NPC. A) Manhattan plot of *P* values derived from the meta‐analysis in two cohorts, where survival analyses were conducted with Cox proportional hazard regression under an additive model adjusted for covariates including age, sex, clinical stage, treatment regimens, and the top five principal components of population structure. The red line represents the significance level of *P* value with correction of multiple comparisons (*P* = 1.56 × 10^−6^). B) Quantile–quantile plot of observed versus expected *P* values. No evidence of inflation was observed (inflation factor λ = 1.102).

**Figure 2:**
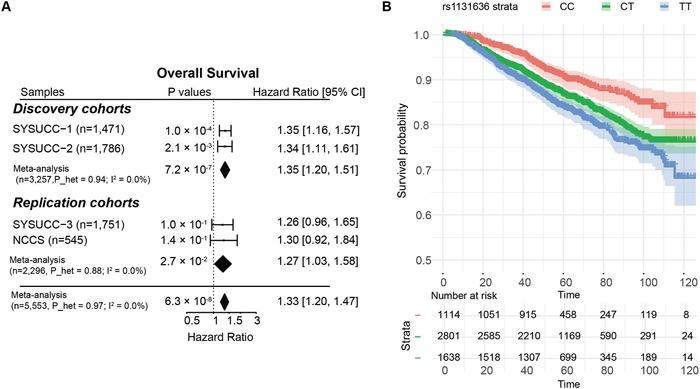
Survival analysis results for rs1131626. A) Forest plot for results from cox proportional hazards regression and meta‐analysis. CI, confidence interval; *P*_het: *P* value from heterogeneity test. B) Kaplan–Meier estimates of overall survival curves in patients with NPC grouped by genotypes of rs1131636 in combined discovery and replication samples. Colors represent patients of different genotypes and shades represent confidence interval for point estimates of survival curves.

After stringent quality control filters in the discovery phase, 1471 patients in the SYSUCC‐1 and 1786 patients in the SYSUCC‐2 with genotypes of 31 870 common SNPs were remained for analyses (Figure S1, Supporting Information). To identify the candidate prognostic markers, survival analyses were first carried out independently in each cohort using Cox proportional hazards regression model, followed by meta‐analysis of the two cohorts under the fixed‐effect model. The quantile‐quantile plot revealed a good match between the distributions of the observed *P* values and the expected ones by chance; and a small genomic control factor indicated a minimal inflation of genome‐wide association significance due to the cryptic population stratification (λ_gc_ = 1.102; **Figure** **1**B). The meta‐analysis revealed that only one SNP rs1131636, located at the 3′‐UTR of *RPA1* on chromosome 17, was significantly associated with overall survival in patients with NPC (HR = 1.35, 95% CI = 1.20–1.51, *P* = 7.21 × 10^−7^; *P*_heterogeneity_ = 0.94, *I* ^2^ = 0%; Figures 1A and **2**A), surpassing the exome‐wide significance (*P* < 1.56 × 10^−6^ as corrected for multiple tests). Moreover, rs1131636 was significantly associated with disease‐free survival (HR = 1.23, 95% CI = 1.11–1.36, *P* = 5.09 × 10^−5^, *I* ^2^ = 0%, *P*_heterogeneity_ = 0.968) and distant‐metastasis‐free survival (HR = 1.23, 95% CI = 1.10–1.38, *P* = 0.0004, *I* ^2^ = 0%, *P*_heterogeneity_ = 0.713), as shown in the Figure S2 and Table S2 (Supporting Information). In addition, rs1131636 was also associated with local‐recurrence‐free survival of patients with NPC, with a borderline significance (HR = 1.20, 95% CI = 1.00–1.44, *P* = 0.0561, *I* ^2^ = 0%, *P*_heterogeneity_ = 0.878; Figure S2 and Table S2, Supporting Information). Patients carrying the risk rs1131636‐T allele tended to have inferior outcomes as compared to those with rs1131636‐[CC] genotype (Figure 2B). The 5 year overall survival was estimated at 90.3% (95% CI = 88.0–92.5%) for patients carrying rs1131636‐[CC] genotype, and 83.4% (95% CI = 82.0–84.9%) for patients with other genotypes. No statistically significant associations between rs1131636 and the baseline clinical and pathological characteristics were consistently observed in both sample collections (Table S3, Supporting Information). In addition, we investigated the relationship between *RPA1* genotypes and EBV IgA titers in a subset of patients with available data (*n* = 1266). We observed that rs1131636‐T was significantly associated with higher antiviral‐capsid‐antigen (VCA) and anti‐early‐antigen IgA titers (*P* = 0.0187 and *P* = 0.032, respectively). Consistently, rs11078676 at an intronic region of *RPA1* was reportedly associated with elevated anti‐VCA IgA titer in healthy Southern Chinese.^[^ ^9^ ^]^ rs1131636 and rs11078676 are within 32 kb distance in *RPA1* and share considerable linkage disequilibrium (*r* ^2^ = 0.11 and *D*′ = 0.75), suggesting that these two SNPs are potentially tagging for a same genetic variant of *RPA1*.

### 2.3 Replication of the Genetic Effect of rs1131636 on NPC Prognosis

To replicate the association between rs1131636 and NPC prognosis, we performed survival analyses in two additional independent samples from Southern China (SYSUCC‐3, *n* = 1751) and Singapore (NCCS, *n* = 545), which revealed consistent trend of association of rs1131636 with overall survival (Figure 2A). Furthermore, meta‐analysis of the two replication cohorts (*n* = 2296) revealed a significant association between rs1131636 and overall survival (HR = 1.27, 95% CI = 1.03–1.58, *P* = 0.0269), consistent with that observed in the discovery (Figure 2A; Table S2, Supporting Information). Moreover, meta‐analysis of all cohorts in the discovery and replication stages (*n* = 5553) revealed a strong association between rs1131636 and overall survival (HR = 1.33, 95% CI = 1.20–1.47, *P* = 6.31 × 10^−8^; Figure 2A; Table S2, Supporting Information), with the best survival estimates for the rs1131636‐[CC] carriers (Figure 2B). Similar unfavorable effects of rs1131636‐T were also observed on other prognosis measurements (disease‐free, distant‐metastasis‐free, and local‐recurrence‐free survivals) in NPC (Figure S2 and Table S2, Supporting Information). Collectively, these observations suggest that patient of the CT/TT genotype might harbor an aggressive tumor subtype leading to poor survival. In addition, we observed similar allele frequency of rs1131636‐T between NPC cohorts and Eastern Asian populations with close geography and ancestry; and interestingly, the highest and lowest frequencies are observed in the European and African/South Asian populations, respectively, which might suggest the allelic heterogeneity and various linkage disequilibrium structures in *RPA1* locus among the populations (Table S4, Supporting Information).

### 2.4 Regulatory Effect of rs1131636 on Gene Expression

As rs1131636 is located at the 3′‐UTR of *RPA1*, we assumed its regulatory effect on gene expression. We performed luciferase reporter assays with a human normal cell line (293T) and NPC cell lines (5–8F and S18). We observed that cells transfected with the rs1131636‐[C] construct had substantially lower luciferase activity than the cells with the rs1131636‐[T] construct, indicating that the variants exhibited regulatory effects on its upstream gene (Figure S3A,B, Supporting Information). In support, eQTL signals were observed at rs1131636 with a *cis* effect on *RPA1* expression (Tables S5 and S6, Supporting Information). Immunohistochemistry analysis revealed higher expression of RPA1 in the samples derived from patients carrying T allele at rs1131636 (genotypes CT or TT) compared to the samples with CC genotypes (Figure S3C, Supporting Information).

### 2.5 RPA1 Modulates the Proliferation, Migration, and Invasion of NPC Cells

**Figure 3:**
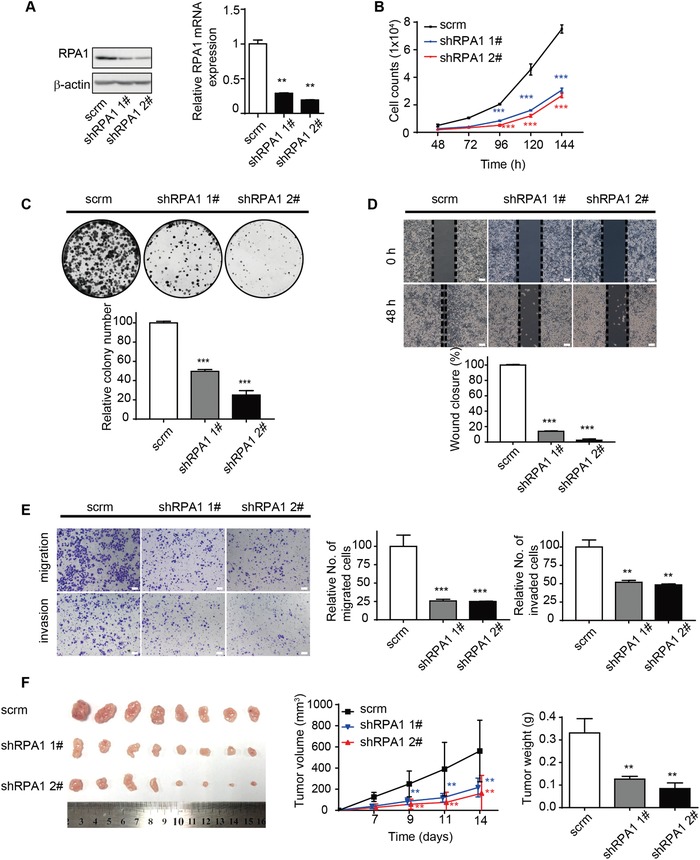
Knockdown of RPA1 inhibited the proliferation, migration, and invasion of S18 NPC cells. A) The down‐regulated expression of RPA1 in S18 cells transduced with lentivirus carrying pLKO.1‐shRPA1 (shRPA1 1# and shRPA1 2#) was verified by using immunoblotting, as compared to that of pLKO.1‐Scrambled‐shRNA (scrm). β‐Actin served as a loading control. The mRNA expression of RPA1 was measured by real‐time PCR analysis and normalized to β‐actin (right). B) Numbers of S18 cells transfected with respective lentivirus construct were determined by averaging the cell numbers in triplicate wells at the indicated time points. C) Colony formation assay was assessed in cell lines with indicated lentivirus construct by crystal violet staining method and the representative images were shown at top. Histogram showed the quantification of colony in three independent experiments (bottom). D) Representative brightfield images for wound‐healing assay in S18 cell lines with indicated lentivirus constructs as being monitored at 0 or 48 h time point (top). Histogram showed the relative wound closure rate of three independent experiments (bottom). Scale bar, 100 µm. E) The migration (left top) and invasion (left bottom) abilities of S18 cells with indicated lentivirus‐constructs were measured by transwell assays without or with Matrigel. Representative images were shown. Scale bar, 100 µm. Histograms showed the fold changes relative to the scrambled cells in three independent experiments. F) Xenograft tumors grown in BALB/c nude mice (*n* = 8 per group) were shown at left panel, which were subcutaneously injected with S18 cells carrying respective lentivirus construct as indicated. The volumes (middle) and the weight (right) of xenograft tumors in nude mice were also measured. All data are shown as mean ± SD from at least three independent experiments. *P < 0.05, **P < 0.01, and ***P < 0.001.

Next, to investigate the functional relevance of RPA1, we manipulated the expression of RPA1 in NPC cells and assess their abilities of proliferation, wound healing, migration, and invasion (**Figure** **3**A; Figure S4, Supporting Information). The knockdown of RPA1 significantly decreased cell proliferation (Figure 3B; Figure S4, Supporting Information) and cell transformation (Figure 3C; Figure S4, Supporting Information). RPA1‐knockdown cells also demonstrated significantly delayed wound healing and reduced abilities to migrate and invade (Figure 3D,E; Figure S4, Supporting Information). In vivo xenograft model revealed that the shRPA1 xenografts with RPA1 knockdown cells had reduced tumor volume and weight, as compared to the control group, indicating that the knockdown of RPA1 significantly inhibited orthotopic tumor formation of NPC cells in mice (Figure 3F). To verify these findings, we introduced exogenous RPA1 in NPC cells and observed that the overexpression of RPA1 conferred aggressive phenotypes to NPC cells (Figures S5 and S6, Supporting Information), in contrast to the effects of the knockdown of RPA1 on NPC cells as abovementioned.

### 2.6 RPA1 Modulates Radiation Sensitivity of NPC Cells

**Figure 4:**
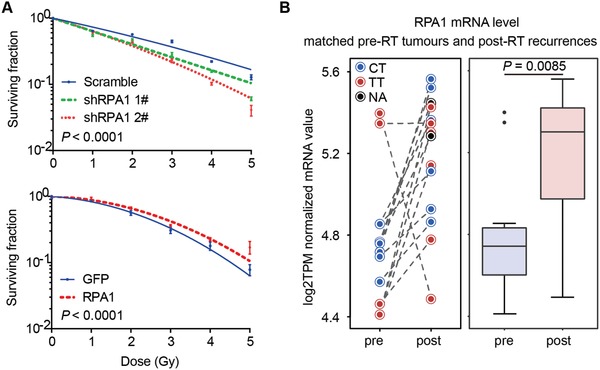
Correlation between RPA1 expression and the radiosensitivity of NPC cells. A) Clonogenic cell survival assay showed the fractions of cells survived from irradiation at indicated doses for S18 cells transduced with lentivirus carrying shRNAs knocking down RPA1 or constructs with exogenous expression of RPA1 or GFP and their respective control constructs. Data points were mean ± SD of the surviving fractions derived from at least three separate experiments. *P* value derived from two‐tailed Student's *t*‐test. B) Left: Longitudinal change in RPA1 gene expression in primary tumors before radiotherapy (pre‐RT; *n* = 10) and recurrent tumors after primary radiotherapy (post‐RT; *n* = 10) from the same patients. Colored circles represent patients with different genotypes of rs1131636 (CT, blue; TT, red; black circle, one patient was not profiled). Right: Box and whisker plot show the median mRNA abundance of both subgroups. Data of longitudinal recurrent samples were available for three patients. *P value derived from Wilcoxon signed‐rank test.

Given the observations that radiosensitization through incremental DNA damage on tumor cells is highly related to NPC remission,^[^ ^10^ ^]^ we performed clonogenic assays to investigate if manipulating RPA1 has any effect on the radiosensitivity of NPC cells. We observed increased radiation sensitivity with knockdown of RPA1, and conversely, radiation resistance with overexpression of RPA1 in NPC cells (**Figure** **4**A). To further validate this finding, we examined the mRNA expression of RPA1 in a subset of primary‐recurrent tumor pairs. The recurrent samples were tumor biopsies following a course of high‐dose radiotherapy upon primary treatment, and thus were truly radioresistant. We observed that the transcription level of RPA1 mRNA was significantly increased in the recurrent samples compared to the paired primary tumors before radiotherapy (Figure 4B). Gene set enrichment analyses (GSEA) in two independent RNA sequencing datasets also revealed a significant correlation between RPA1 and transcriptional expression of genes involved in repairing DNA damage including homologous recombination (in‐house data: *n* = 87, NES = 1.93, *P* < 0.0001; GSE102349: *n* = 113, NES = 1.63, *P* = 0.0299) and nucleotide excision repair (in‐house data: NES = 1.99, *P* < 0.0001; GSE102349: NES = 1.96, *P* < 0.0001) pathways (Figure S7, Supporting Information), thereby supporting this functional role of RPA1.

### 2.7 miR‐1253 Targeted rs1131636 and Suppressed the Proliferation and Migration of NPC Cells

**Figure 5:**
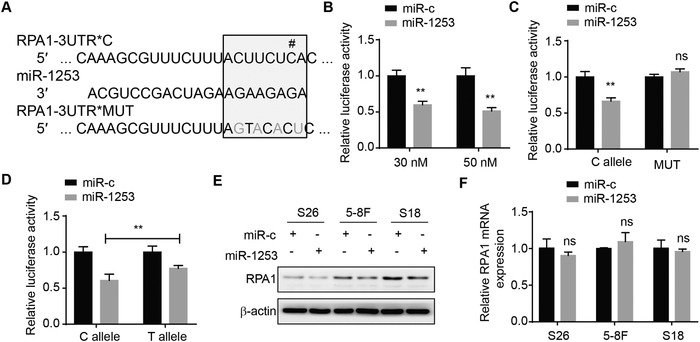
miR‐1253 targeted rs1131636 and suppressed expression of RPA1. A) Sequences were shown for miR‐1253 (middle), the predicted targeting site of miR‐1253 on the 3′‐UTR of RPA1 (top; RPA1‐3UTR*C; # indicates position of rs1131636) and a negative control with four mutations as indicated (bottom; RPA1‐3UTR*MUT). The miR‐1253 seed region was boxed. B) Luciferase activities in HEK293T cells transfected with increasing amounts of synthetic miR‐1253 mimics or scrambled miRNAs negative control (miR‐c). C) Luciferase activities in HEK293T cells cotransfected with RPA1‐3UTR*C or *MUT reporters and miR‐1253 mimics or scrambled miRNAs as negative control (miR‐c). D) Luciferase activities in HEK293T cells transfected with the psiCHECK2 constructs with either C or T allele at rs1131636 in *RPA1* 3′‐UTR fragment, together with miR‐1253 mimics or scrambled miRNAs negative control (miR‐c). E) Western blotting showed the expression of RPA1 and β‐actin as control in the S26, 5–8F and S18 cells transfected with either miR‐1253 mimics or scrambled miRNAs negative control (miR‐c). F) The mRNA expression of RPA1 was measured by real‐time PCR analysis in S26, 5–8F and S18 transfected with miR‐1253 mimics and scrambled miRNAs as negative control (miR‐c) and normalized to β‐actin. All data B–D,F) are shown as mean ± SD from at least three independent experiments. *P < 0.05, **P < 0.01, and ***P < 0.001.

To further investigate how rs1131636 regulates upstream *RPA1*, we identified that miR‐1253 might target a miRNA binding site at the 3′‐UTR of *RPA1* (717–723 bp starting from the stop codon; **Figure** **5**A), using TargetScan. Luciferase reporter assays showed that the luciferase activity of *RPA1* 3′‐UTR was significantly reduced in the 293T cells transfected with miR‐1253 mimics compared to the control group in a dose‐dependent manner (Figure 5B), and the inhibitory effect of miR‐1253 was abolished when mutations were introduced to the targeting site (Figure 5C). Moreover, luciferase reporter assays also revealed that the transfection of miR‐1253 mimics reduced the luciferase activity of *RPA1* 3′‐UTR with greater inhibitory effect on the construct of rs1131636‐C allele (0.57‐fold) than rs1131636‐T allele (0.77‐fold; Figure 5D). These results suggested that miR‐1253 binds to *RPA1* 3′‐UTR, and the binding affinity is regulated by different alleles of rs1131636. Furthermore, the transfection of miR‐1253 led to downregulation of RPA1 at the protein level but not mRNA level in NPC cells (Figure 5E,F), suggesting that miR‐1253 binds to *RPA1* 3′‐UTR to hinder its protein translation. This is corroborated by the observations that miR‐1253 inhibited the proliferation and migration of NPC cells (Figure S8, Supporting Information), which was consistent with the phenotypes observed in the cells with knockdown of RPA1 (Figure 3; Figure S4, Supporting Information). In addition, we observed the presence of miR‐1253 in NPC tissues and human cell lines including 5–8F, S18, and 293T (Figure S9, Supporting Information).

## 3 Discussion

With the bioinformatic analyses and biological evidence in current study, we identified a novel germline polymorphism of *RPA1* gene (rs1131636) conferring tumor progression and therapeutic resistance in NPC, which ultimately affects the survival of patients with NPC. Patients with CC genotype at rs1131636 (account for 20% of all NPC patients) had a better survival due to superior disease control, as compared to patients with CT or TT genotype who might receive further intensified systematic therapy. We thus propose that rs1131636 at *RPA1* could be a promising germline biomarker to predict therapeutic efficacy and outcome for patients with NPC. Our findings, together with other similar studies^[^ ^11^ ^]^ support a provocative notion that germline polymorphisms may serve as potential biomarkers for predicting tumor biology and precision medicine.

Given that rs1131636 is located at the 3′‐UTR of *RPA1*, we provided a series of experimental evidence showing that rs1131636 has regulatory effect on the expression of upstream *RPA1*, which thereby might contribute to the progression of NPC. RPA1 (or p70), together with RPA2 (p34) and RPA3 (p14), forms the heterotrimeric replication protein A (RPA) complex,^[^ ^12^ ^]^ which is essential for maintenance of telomeres^[^ ^13^ ^]^ and regulating cell cycle,^[^ ^14^ ^]^ as well as for DNA replication, repair, and recombination.^[^ ^15^ ^]^ It has been reported that mutations in *RPA1* induced defective DNA double‐strand break repair, chromosomal instability and development of tumor in mice.^[^ ^16^ ^]^ Our study revealed that *RPA1* could promote cell proliferation, migration and invasion in NPC cells with both in vitro and in vivo experiments, which is consistent with previous observations in other cancers.^[^ ^17^ ^]^ Given that EBV infection is a well‐known hallmark of NPC in endemic regions, we and others observed correlations between *RPA1* polymorphisms (rs1131636 and rs11078676) and IgA titers against EBV antigens in patients with NPC and healthy individuals, respectively.^[^ ^9^ ^]^ These findings implicate that *RPA1* might play an important role in regulating the activation of EBV and host immune response against its infection,^[^ ^18^ ^]^ while the underlying mechanisms are yet to be addressed. In addition, previous studies have demonstrated the expression of RPA1 and RPA2 are potential prognostic factors in multiple cancers,^[^ ^17^, ^19^ ^]^ and likewise, RPA3 has been associated with survival of patients with gastric cancer,^[^ ^20^ ^]^ hepatocellular cancer,^[^ ^21^ ^]^ and NPC.^[^ ^22^ ^]^ These findings suggest that RPA1 and other subunits of RPA complex might be important modulators for the progression of several human cancers.

Radiotherapy is the primary and curative treatment for NPC.^[^ ^23^ ^]^ Our data confirm that RPA1 plays a role in modulating the radiosensitivity of NPC cells, whereby overexpression of RPA1 leads to radioresistance. This is consistent with a very recent finding in another NPC cell line,^[^ ^24^ ^]^ as well as the finding in esophageal cancer.^[^ ^25^ ^]^ The correlation of *RPA1* to the genes involved in crucial DNA repair pathways as revealed by transcriptome analysis would suggest that NPC tumors with higher expression or activation of RPA1 might result in a higher repair fidelity of radiation‐induced DNA damage leading to radioresistance.^[^ ^26^ ^]^ Next, we identified that miR‐1253 can target the seeding region spanning rs1131636 at the 3′‐UTR of *RPA1*, which suppresses the protein expression of RPA1, but not transcription. Higher inhibitory effect of C allele of rs1131636 was also observed as compared with its counterpart T allele, suggesting that the accessibility of miRNA to the locus harboring rs1131636‐C is higher and thus exerts stronger inhibition on RPA1 translation. Therefore, it is plausible that the favorable outcomes of NPC patients with CC‐genotype may be linked to increased tumor radiosensitivity and less aggressiveness due to reduced RPA1 expression, as opposed to patients with the CT or TT genotype. In addition, miR‐1253 has been reported as a biomarker for certain types of cancers, and a regulatory miRNA binding to 3′‐UTR of gene targets to promote tumorigenesis.^[^ ^27^ ^]^ Nevertheless, it is unclear that how miR‐1263 is regulated, although sponging of miR‐1253 by circular RNAs has been demonstrated.^[^ ^28^ ^]^

Our study has a number of strengths. Foremost, to the best of our knowledge, this is the first study to investigate associations between genetic variants and NPC prognosis, with a substantially large sample size and maximal coverage of genes by far. Second, the distributions of clinical and pathological characteristics are consistent with previous reports, revealing unbiased selection. Third, our discovery withstood a strict threshold for statistical significance that is corrected for multiple tests, and the clinical associations remained significant after adjustment for known prognostic factors. We note that the hazard ratios on survival estimates appear to be less significant in the replication stage compared to the discovery stage, likely reflecting the effect of Winner's curse,^[^ ^29^ ^]^ which could be attributed to different genomic structures of linkage disequilibrium underlying causal variants, heterogenous factors contributing to disease control and survival outcomes, and smaller sample sizes in the replication cohorts. Taken together with our consistent observations among all cohorts and the experimental evidence, these assure that the likelihood of a potential false discovery is low.

Our study also has some limitations. First, our findings are based on a retrospective study in population of Southern Chinese descendants, further replications in more population and with prospective design are needed to confirm the significance of rs1131636 as a germline biomarker to predict NPC prognosis, aiding in stratifying patient participants with more aggressive behaviors and poor survival for clinical trials. Second, we acknowledge that further studies should be performed to fine map the genetic variations within *RPA1* gene locus and explore their regulatory potentials on RPA1 expression, as well as the exact role of RPA1 as a potential target of radiosensitizer for treatment of NPC.

## 4 Experimental Section

##### Patient Recruitment and Follow‐Up

A two‐stage design including discovery and replication was adopted for this study. At the discovery stage, patients with NPC from two consecutive cohorts were recruited at the Sun Yat‐sen University Cancer Centre (SYSUCC; Guangzhou, China); the first cohort of 1485 patients was enrolled from March 3, 2003 to December 28, 2007 (SYSUCC‐1), and the second cohort of 1791 patients was enrolled from January 1, 2008 to April 25, 2012 (SYSUCC‐2). At the replication stage, two more independent cohorts were recruited, including 1751 patients from SYSUCC between April 22, 2008 and June 1, 2015 (SYSUCC‐3), and 545 patients from the National Cancer Centre Singapore (Singapore) between January 1, 2008 and June 14, 2018 (NCCS). For evaluation of gene expression, additional 132 and 10 patients were recruited at SYSUCC (January 11, 2011 to November 28, 2013) and NCCS (January 1, 2008 and June 14, 2018), respectively.

All subjects were histologically diagnosed with NPC and subsequently treated at the recruitment sites. Individuals with history of cancer, radiotherapy, chemotherapy, or any other antitumor therapy prior to first diagnosis were excluded. Clinical staging of NPC was determined according to the 7th edition of the International Union Against Cancer (UICC) and American Joint Committee on Cancer (AJCC) staging system.^[^ ^30^ ^]^ The follow‐up data were collected at 3 month intervals for the first two years and six monthly thereafter. The date of last follow‐up for the discovery samples (SYSUCC‐1 and ‐2) was August 22, 2016; and for the replication cohorts, it was March 15, 2019 for SYSUCC‐3, and February 25, 2019 for NCCS. Local recurrence was confirmed by fiberoptic endoscopy, MRI and biopsy, whereas distant metastases were diagnosed by clinical symptoms, physical examination, and imaging methods include computed tomography (CT), bone scan, abdominal sonography, and/or ^18^F‐fluorodeoxyglucose positron emission tomography‐CT (^18^F‐FDG‐PET‐CT). Serum immunoglobulin A (IgA) antibodies to Epstein–Barr virus (EBV) capsid antigen (IgA‐VCA) and to the EBV early antigen (IgA‐EA) were available for 1251 individuals from SYSUCC‐1 cohort. Written informed consent was obtained from all patients enrolled in this study, and the study was approved by the local ethics committees at SYSUCC and NCCS.

##### Genotyping and Quality Control

EDTA‐anticoagulant peripheral whole blood sample was obtained from each patient at the time of diagnosis and before any treatment. Genomic DNA sample was extracted from the blood sample using Qiagen blood midi or maxi kits (Qiagen, Duesseldorf, Germany). For the two cohorts in the discovery stage (SYSUCC‐1 and SYSUCC‐2), genome‐wide genotyping of 246173 SNPs was done at the Genome Institute of Singapore (Singapore) and the CapitalBio Technology Co., Ltd (Beijing, China), by using Infinium HumanExome BeadChips (Illumina, San Diego, California, USA), which contains a total of 246173 SNPs and mostly exonic SNPs of rare to low frequency in population. As for SYSUCC‐3 cohort in the replication stage, the genotypes of candidate SNP were retrieved from the genome‐wide data, which were obtained by using Infinium Global Screening Array BeadChip (Illumina, San Diego, California, USA) followed by imputation using the IMPUTE2 program (version 2.3.2)^[^ ^31^ ^]^ with default parameters. For the NCCS cohort in the replication stage, the genotypes of the candidate SNP were directly typed by TaqMan assay (Applied Biosystems, Waltham, Massachusetts, USA) on CFX384 Real‐Time system (Bio‐Rad, Hercules, California, USA) according to the manufacturer's instruction.

Stringent quality control filters were applied to remove genotype markers with poor quality or low frequency in both cohorts in the discovery stage, using the PLINK program (version 1.07).^[^ ^32^ ^]^ SNPs with genotyping successful rate of less than 95% were excluded, as were SNPs of nonautosomal, those with minor allele frequency (MAF) less than 1%, and those deviating from Hardy–Weinberg equilibrium (HWE) test (*P* < 1 × 10^−6^). Similarly, quality control filters were done among individuals. Samples with low genotyping efficiency less than 95% and with discordant genders to their recorded ones (implying possible unintended technical error in the sample processing) were excluded; moreover, samples showing extremes of heterozygosity (inbreeding coefficient estimate *F* < –0.01, indicating possible cross‐contamination) were also excluded; furthermore, for pairs of individuals with cryptic relatedness according to identity‐by‐descent (IBD) calculation implemented in PLINK (possibly due to unintended technical error or biological relatives). Lastly, ethnic outliers as revealed by principal component analysis (PCA) of genetic ancestry were removed. Consequently, a total of 3257 samples and 31870 SNPs passing quality control were subjected for further analyses (Figure S1, Supporting Information).

##### Cell Culture

The human embryonic kidney cells 293T (HEK293T) and two NPC cell lines (S18 and 5–8F) were cultivated in Dulbecco's modified Eagle medium (DMEM; Gibco, Grand Island, NY, USA) supplemented with 10% fetal bovine serum (FBS; Gibco, Grand Island, NY, USA) and maintained under standard cell culture conditions at 37 °C in a water‐saturated atmosphere of 5% CO_2_. HEK293T was purchased from American Type Culture Collection (ATCC). The two NPC cell lines, 5–8 F and S18, were established and maintained at the SYSUCC. 5–8 F was characterized as a subclone with high tumorigenic and metastatic ability from the parental cell line SUNE‐1, which was derived from a patient with NPC treated at SYSUCC.^[^ ^33^ ^]^ S18 was a clone, showing great migration and invasion ability, picked up from a single cell colony of CNE‐2, which was derived from a patient with NPC from Southern China.^[^ ^34^ ^]^ Both 5–8 F and S18 have lost their EBV genome/episomes upon in vitro propagations and became EBV negative.^[^ ^35^ ^]^

##### RNA Extraction and Reverse Transcription (RT) PCR Analysis

Total RNA was isolated using Trizol reagent (Invitrogen, Carlsbad, CA, USA) and reverse transcription was performed using oligo (dT) priming and M‐MLV Reverse Transcriptase according to the manufacturer's instructions (Promega, Madison, WI, USA). Real‐time quantitative PCR was performed in the StepOne Real‐Time PCR System (Applied Biosystems, Waltham, Massachusetts, USA) using primer pairs according to gene of interest (Table S7, Supporting Information) and SYBR Green as DNA dye (Takara, Tokyo, Japan). miRNA level was quantified by stem‐loop RT‐qPCR method using Bulge‐Loop hsa‐miR‐1253 Primer Set (RIBOBIO, China). Then, the PCR products were cloned into pMD19‐T Vector (TAKARA, Japan). The plasmids contained miRNA was determined using Sanger sequencing.

##### Luciferase Assays

DNA fragments containing *RPA1* 3′‐UTR with either C (RPA1‐3UTR*C) or T allele (RPA1‐3UTR*T) at rs1131636 were obtained by using reverse transcribed (RT)‐PCR and mRNA extracted from NPC cells as template (5–8F cell line; primer information is listed in Table S7 in the Supporting Information). To examine the targeting effect of miR‐1253 on *RPA1* 3′‐UTR region spanning rs1131636 as predicted by using TargetScan,^[^ ^36^ ^]^ a mutant construct of *RPA1* 3′‐UTR was obtained by replacing the seeding sequence with 5′‐CAAAGCGTTTCTTTAgTaCaCtCTTCAATTAATGC‐3′ (RPA1‐3UTR*MUT). Each of the DNA products was separately subcloned into a luciferase reporter plasmid at the downstream of luciferase coding region (psiCHECK2; Promega, Madison, WI, USA) and the sequence validity was confirmed by Sanger sequencing. Next, 5 × 10^4^ cells were seeded into each well of 24‐well plate and simultaneously transfected with respective construct and either miR‐1253 mimics or scrambled control miRNAs (GenePharma, Shanghai, China) using lipofectamine 2000 (Invitrogen, Carlsbad, CA, USA). After being cultured for 36 h, luciferase activity of the cells was measured with a Dual‐Luciferase Assay Kit (Promega, Madison, WI, USA) according to the manufacturer's protocol.

##### Stable Knockdown and Overexpression of RPA1 in Cells

Lentiviral expression system was deployed to manipulate the expression of RPA1 in cells. For knockdown with shRNAs, double‐strand oligos (GenePharma, Shanghai, China; sequences are provided in Table S7 in the Supporting Information) were inserted to lentiviral vector pLKO.1 following the protocols from Addgene (https://www.addgene.org/). For overexpression, full length of human *RPA1* cDNA was obtained using RT‐PCR and mRNA extracted from NPC cells as template (5–8F; primer information is listed in Table S7 in the Supporting Information), and subsequently cloned into the pCDH vector (pCDH‐Flag‐RPA1); and as a negative control, the gene encoding green fluorescent protein (GFP) was also constructed into the pCDH vector separately (pCDH‐GFP). For lentivirus production, lentiviral construct was co‐transfected with packaging vectors ∆8.9 and VSVG into HEK293T cells using Lipofextamine 2000 (Invitrogen, Carlsbad, CA, USA) following the manufacturer's instructions. Viral supernatant was collected at 48 h post transfection and filtered through 0.45 µm PVDF filters. For transfection, lentivirus with polybrene (8 µg mL^−1^; Sigma, San Antonio, TX, USA) were added to the cells; and subsequently the cells were maintained for 48 h, followed by selection of infected cells with 2 µg mL^−1^ puromycin (Solarbio, Beijing, China) for one week.

##### Immunoblotting Analysis

Proteins were extracted from the whole cell lysates as described previously.^[^ ^37^ ^]^ In brief, whole cell lysates were prepared in cold low‐salt lysis buffer with protease inhibitor cocktail (Roche, Basel, Switzerland), followed by centrifugation at 12 000 rpm for 5 min at 4 °C. Proteins in the supernatant were then subjected to sodium dodecyl sulfate‐polyacrylamide gel electrophoresis and transferred to PVDF membranes (Millipore, Billerica, MA, USA). Membranes were subsequently blocked with 5% defatted milk and incubated with primary anti‐RPA1 antibody (sc‐28304; Santa Cruz, CA, USA), antiflag antibody (F1804; Sigma‐Aldrich, St. Louis, MO, USA) and antiactin antibody (A1978; Sigma‐Aldrich, St. Louis, MO, USA), separately.

##### Immunohistochemistry

Paraffin‐embedded section was deparaffinated and rehydrated, followed by antigen retrieval and blockage with 5% normal goat serum and subsequent incubation with primary antibody against RPA1 (Santa‐Cruz, sc‐28304) at 4 °C for overnight. Immunostaining were performed with horseradish peroxidase (HRP) conjugates by using Dako REAL EnVision Detection System. Staining condition were evaluated independently by two pathologists. Protein expression level was analyzed according to staining frequency and intensity. The staining frequency of the protein was semiquantitatively scored based on the percentage of positive cells at four quantiles, which was multiplied by intensity scores as 0, 1, 2, and 3 for no, weak, intermediate, and strong staining, respectively, to obtain immunohistochemical scores.

##### Whole Exome Sequencing and Genotype Calling

Genomic DNA sample was subjected for library construction using SureSelectXT Human All Exon + UTRs kit (Agilent Technologies, Santa Clara, CA, USA), followed by sequencing with a paired‐end 2 × 125 bp protocol on a HiSeq instrument (Illumina, San Diego, CA, USA). Sequencing data was aligned to the human reference genome (GRCh37/hg19) using the BWA software.^[^ ^38^ ^]^ Subsequently, the GATK suite was used for base quality score recalibration, realignment of insertion/deletions, and variant calling, according to the GATK Best Practices recommendations.^[^ ^39^ ^]^ The genotypes of rs1131636 for the 132 patients from SYSUCC and 10 patients from NCCS were retrieved from the whole exome data.

##### RNA Sequencing

RNA sample preparation and RNA sequencing were performed by following the standard protocols provided in the commercial kits, elsewhere stated. Total RNA was extracted from NPC tumor tissue (*n* = 87) by using the RNeasy Mini Kit (Qiagen, Duesseldorf, Germany). Ribosomal RNAs were depleted using Ribo‐Zero Magnetic Kit (Illumina, San Diego, California, USA). Paraffin embedded (FFPE) primary‐recurrent nasopharyngeal tumor pairs were retrieved in the ten patients from NCCS and were macrodissected for RNA extraction. Total RNA was extracted using AllPrep DNA/RNA FFPE Kit (Qiagen, Duesseldorf, Germany) following the protocol. Library preparation was performed using TruSeq RNA prep kit (Illumina, San Diego, California, USA). Libraries with different adaptors were pooled and sequenced using the HiSeq 1500 or Hiseq X Ten instruments, yielding approximately 25 million pair‐ended 125 or 150 bp reads per sample.

Sequencing data in fastq format was obtained from the instruments, after data conversion from BCL format using the bcl2fastq software (version 2.18; Illumina, San Diego, California, USA). The adaptors in the raw fastq data were then trimmed using cutadapt (version 1.11).^[^ ^40^ ^]^ Read pairs with low quality were removed, as were reads with high quality but could be aligned to ribosome RNAs using Bowtie 2 (version 2.3.4).^[^ ^41^ ^]^ Reads after these filters were realigned to reference human genome (hg19).^[^ ^42^ ^]^ Finally, the read count for each gene was quantitated using HTseq (version 0.9.1),^[^ ^43^ ^]^ and expression level of each gene was normalized as transcripts per million reads (TPM) or normalized to log 2 transformed transcripts per kilobase million (log 2 TPM) followed by quantile normalization for downstream analysis.

##### Cell Proliferation, Migration, and Invasion Assays

For cell proliferation assay, 1 × 10^3^ cells were seeded into each well of 96‐well plate. Cell number and viability were determined at the indicated time using Trypan blue exclusion under a light microscope. For colony forming assay, 1 × 10^3^ cells were seeded in each well of 6‐well plate and cultured for two weeks. Cells were fixed with methanol and stained with 0.5% crystal violet for 15 min at room temperature and subsequently the colonies of each well were enumerated under a light microscope. Wound healing assays were performed using two well silicone inserts (ibidi, Germany) according to the manufacturer's instructions. Images were captured by using an inverted microscope (IX73; Olympus, Tokyo, Japan) and the migration ability of the cells was calculated as the area measured in pixels between the edges of the scratching at the indicated time point in relative to that of the starting time point using ImageJ software (version 1.32, https://imagej.nih.gov/ij/). Cell migration and invasion assays were performed using Transwell chambers (8 µm pores; Corning, NY, USA) precoated with or without matrigel (Corning, NY, USA). 5 × 10^4^ cells in serum‐free medium were plated into the top chamber of each insert, whereas 600 µL DMEM containing 10% FBS was added to the lower chambers. Cells were then stained with 0.5% crystal violet after incubation for 20 h and subsequently stained cells were enumerated under a light microscope. Each test was performed in triplicate.

##### In Vivo Xenograft Tumor Model

Four‐week‐old male BALB/c nude mice were purchased from Beijing Vital River Laboratory Animal Technology (Beijing, China). Mice were randomly divided into experimental groups. Cell suspension containing 1 × 10^6^ of S18 cells per 100 µL were subcutaneously injected into dorsal flank of the nude mice (ten mice per group). Tumor size was measured every three days. Finally, mice were sacrificed, and tumors were collected for further analysis. Tumor volume was calculated by the formula as *V* = length × width^2^ × 0.52.^[^ ^44^ ^]^ All animal experiments were performed under protocols approved by the Institutional Animal Care and Use Committee of Sun Yat‐sen University.

##### Clonogenic Cell Survival Assay

The clonogenic assays were performed according to a modified protocol as previously described.^[^ ^45^ ^]^ Briefly, 1 × 10^3^ of S18 cells were seeded in each well of a 6‐well plate and cultured overnight, followed by irradiation at 0, 1, 2, 3, 4, and 5 Gy using a Rad Source R2000 X‐ray irradiator (1.1 Gy min^−1^, 160 kV, 25 mA, 0.3 mm copper filters; Rad Source Tech, USA). After 8–10 days of incubation, cells were rinsed twice with phosphate‐buffered saline (PBS), and then fixed with methanol and stained with 0.5% crystal violet. The stained colonies were enumerated. The plating efficiency (PE) was calculated as the ratio of the number of colonies counted to the number of cells seeded, multiplying by 100. The surviving fractions (SF) were then determined by dividing the PE of the treated cells by the PE of the controls (with irradiation dose of 0 Gy) and multiplying by 100.

##### Statistical Analysis

Patient clinical and pathological characteristics were summarized either as numbers and percentages of patients for categorical data or as median and range of values for continuous data. The primary endpoint for this study is overall survival (OS), defined as time from the first treatment after primary diagnosis to death of any cause. Other secondary endpoints included and their definitions are as following: disease‐free survival (DFS) defined as the time from first treatment to the date of first recurrence or death of any cause; distant metastasis‐free survival (DMFS) defined as the time from first treatment to the date of first distant metastasis or death of any cause, and local recurrence‐free survival (LRFS) defined as the time from first treatment to the date of first loco/regional recurrence or death of any cause. Patients who were alive and/or not recorded progression were censored at the date of last follow‐up.

The association between genetic variants and survival was performed using Cox proportional hazards regression analyses under additive genetic model, with adjustment for known prognostic covariates, including age, sex, clinical stage, treatment regimen, and population. Genotypes for each SNP were treated as variables of 0, 1, and 2 representing the homozygote of minor allele, heterozygote, and the homozygote of major allele, respectively. Population structure was estimated by using PCA, and the first five principal components were included in the regression analyses. The Cox model estimated hazard ratio (HR) and 95% confidence interval (CI) for individual sample and meta‐analysis between samples was performed under fixed‐effect model using R package survival (version 2.44‐1.1)^[^ ^46^ ^]^ and Metafor (version 2.0‐0).^[^ ^47^ ^]^ Bonferroni correction was used to adjust for multiple comparisons. Survival curves were estimated using the Kaplan–Meier method. The associations between the variants of interest and the clinical and pathological characteristics were determined using two‐tailed Chi‐square test for a categorical variable and two‐tailed Student's *t*‐test for a continuous variable. Linear regression models were applied to regress rank‐transformed IgA titers against VCA and EA, separately, on the SNP genotypes under additive model, jointly fitted with age, sex, stage, and first five genetic principal components as covariates. The expression quantitative trait loci (eQTL) surveys were conducted against GTEx portal^[^ ^48^ ^]^ (release V7, https://www.gtexportal.org/home/) and Blood eQTL browser^[^ ^49^ ^]^ (https://genenetwork.nl/bloodeqtlbrowser/). Student's *t*‐test was applied to compare the difference between two groups, using GraphPad Prism 7 (version 7.00; GraphPad Software, USA). Wilcoxon signed‐rank test (paired samples) implicated in R program was used to compare gene expression between the paired preradiotherapy tumors and postradiotherapy recurrences, with a two‐sided *P* < 0.05 for statistical significance.

The clonogenic survival curves were fitted to a linear‐quadratic model and compared using the extra sum‐of‐squares *F*‐test. Gene set enrichment analysis was performed using the GSEA desktop application (version 3.0; Broad Institute at MIT, USA). The gene sets implemented were derived from the Kyoto Encyclopedia of Genes and Genomes (KEGG) pathway database (c2.cp.kegg.v6.2.symbols.gmt, 186 gene sets), which was collected in the Molecular Signatures Database (MSigDB; version 6.2).^[^ ^50^ ^]^ One thousand permutations were taken in GSEA. The input phenotypes were the high or low expression levels of RPA1 normalized to the mean of RPA1 expression among all the tested samples. For the mRNA sequencing data retrieved from Gene Expression Omnibus database (series GSE102349; *n* = 113), same pipeline was applied for above data processing. *P* < 0.05 was considered to indicate a significant difference, unless otherwise stated. The key raw data have been deposited in the Research Data Deposit (RDDB2020000816; https://www.researchdata.org.cn).

## Conflict of Interest

M.L.K.C. reports personal fees from Astellas, personal fees from Janssen, grants and personal fees from Ferring, nonfinancial support from Astrazeneca, personal fees and nonfinancial support from Varian, grants from Sanofi Canada, grants from GenomeDx Biosciences, nonfinancial support from Medlever, nonfinancial support from PVMed, outside the submitted work. D.Y.M.L. is the director of Take2, DRA, and consultant of Grail and Decheng Capital, and holds equity of Grail, Take2, DRA. E.P.H. reports Speaker's honoraria from Merck Sharp and Dohme and Merck Serono, participates as Consultant/Advisory Boards for Merck Sharp and Dohme, and received Research funding (institution) from Merck Sharp and Dohme, Pfizer. A.K.C.C. is the director of Take2, DRA, and consultant of Grail, and holds equity of Grail, Take2, DRA. All remaining authors have declared no conflicts of interest.

## Author Contributions

Y.‐M.G., J.‐R.C., Y.‐C.F., M.L.K.C., and Y.Z. contributed equally to this work. J.‐X.B., Y.‐X.Z., J.L., and H.‐Q.M. jointly supervised this work.

## Supporting information

Supporting Information
