# Supplementary material for: Germline Polymorphisms and Length of Survival of Nasopharyngeal Carcinoma: An Exome‐Wide Association Study in Multiple Cohorts
Source: Adv Sci (Weinh). 2020 Mar 20;7(10):1903727. doi: 10.1002/advs.201903727 (PMC7237860; doi:10.1002/advs.201903727)
Supplement: Supplementary file 1 — Supporting Information [file ADVS-7-1903727-s001.pdf]

Copyright WILEY-VCH Verlag GmbH & Co. KGaA, 69469 Weinheim, Germany, 2020.

ADVANCED SCIENCE
Open Access

Supporting Information

for *Adv. Sci.,* DOI: 10.1002/advs.201903727

Germline Polymorphisms and Length of Survival of Nasopharyngeal Carcinoma: An Exome-Wide Association Study in Multiple Cohorts

Yun-Miao Guo, Jie-Rong Chen, Yan-Chun Feng, Melvin L. K. Chua, Yanni Zeng, Edwin Pun Hui, Allen K. C. Chan, Lin-Quan Tang, Lin Wang, Qian Cui, Hui-Qiong Han, Chun-Ling Luo, Guo-Wang Lin, Yan Liang, Yang Liu, Zhong-Lian He, Yu-Xiang Liu, Pan-Pan Wei, Chu-Jun Liu, Wan Peng, Bo-Wei Han, Xiao-Yu Zuo, Enya H. W. Ong, Eugenia L. L. Yeo, Kar Perng Low, Gek San Tan, Tony K. H. Lim, Jacqueline S. G. Hwang, Bo Li, Qi-Sheng Feng, Xiaojun Xia, Yun-Fei Xia, Josephine Ko, Wei Dai, Maria L. Lung, Anthony T. C. Chan, Dennis Y. M. Lo, Mu-Sheng Zeng, Hai-Qiang Mai, Jianjun Liu,* Yi-Xin Zeng,* and Jin-Xin Bei**

Copyright WILEY-VCH Verlag GmbH & Co. KGaA, 69469 Weinheim, Germany, 2020.

Supporting Information

Germline polymorphisms and length of survival of nasopharyngeal carcinoma: an exome-wide association study in multiple cohorts

*Yun-Miao Guo, Jie-Rong Chen, Yan-Chun Feng, Melvin L. K. Chua, Yanni Zeng, Edwin Pun Hui, Allen K. C. Chan, Lin-Quan Tang, Lin Wang, Qian Cui, Hui-Qiong Han, Chun-Ling Luo, Guo-Wang Lin, Yan Liang, Yang Liu, Zhong-Lian He, Yu-Xiang Liu, Pan-Pan Wei, Chu-Jun Liu, Wan Peng, Bo-Wei Han, Xiao-Yu Zuo, Enya H. W. Ong, Eugenia L. L. Yeo, Kar Perng Low, Tony K. H. Lim, Jacqueline S. G. Hwang, Bo Li, Qi-Sheng Feng, Xiao-Jun Xia, Yun-Fei Xia, Josephine Ko, Wei Dai, Maria L. Lung, Anthony T. C. Chan, Dennis Y. M. Lo, Mu-Sheng Zeng, Hai-Qiang Mai, Jianjun Liu, Yi-Xin Zeng, Jin-Xin Bei**

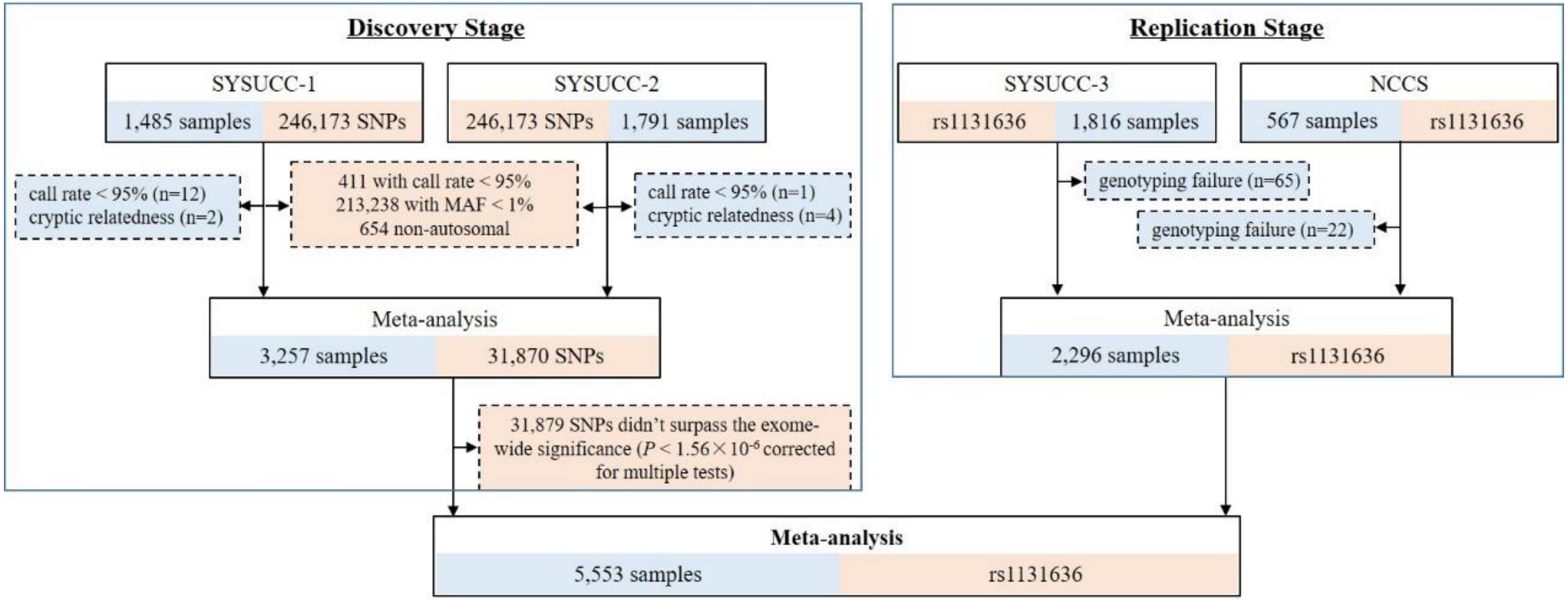

Figure S1. Quality control for study collections. SYSUCC-1/2/3, patient recruitment at Sun Yat-sen University Cancer Centre; NCCS, patient recruitment at National Cancer Centre of Singapore; MAF, minor allele frequency.

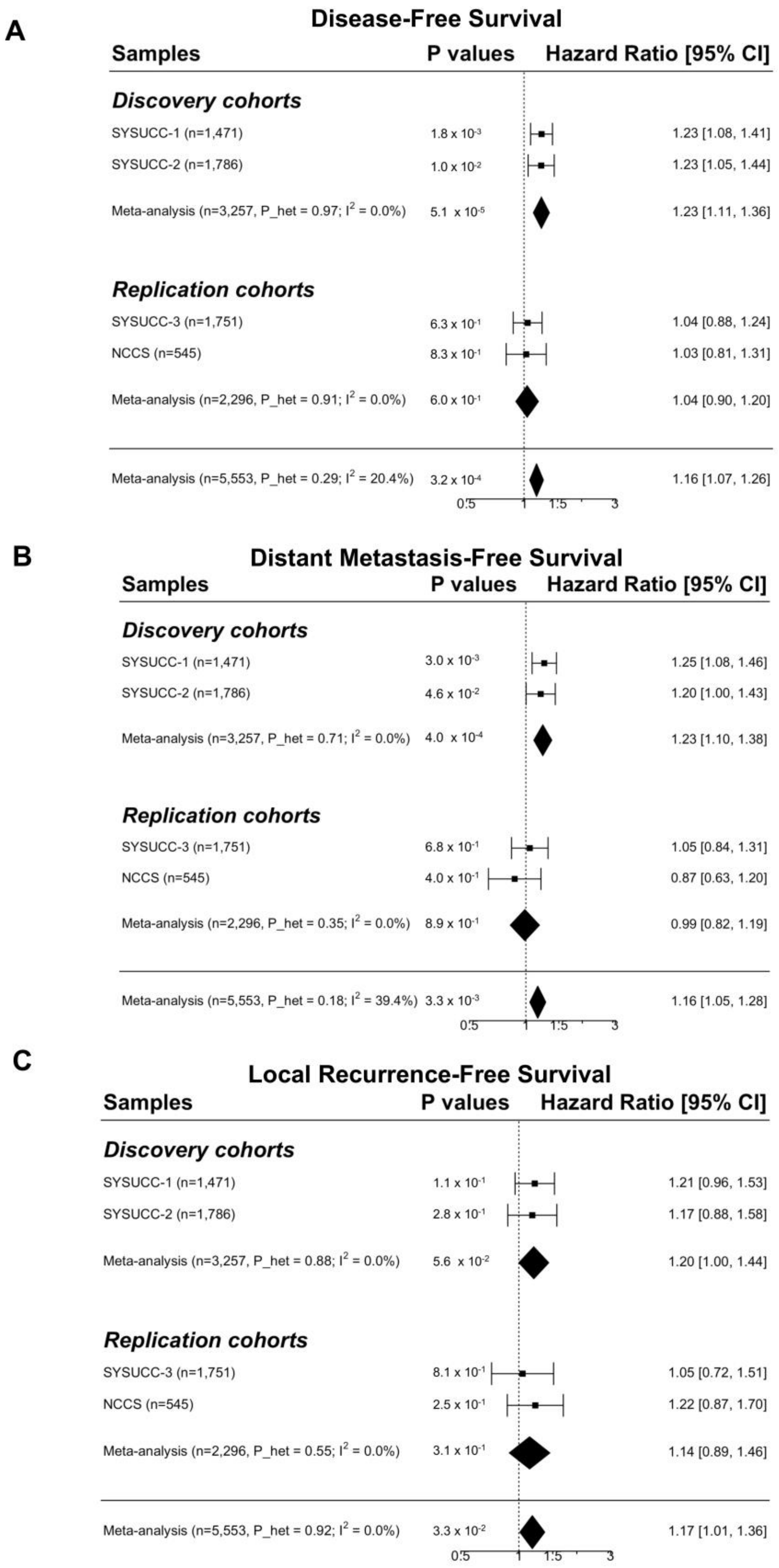

Figure S2. Forest plots of the association results for survival status. (A) disease-free survival, (B) distant metastasis-free survival, and (C) local recurrence-free survival other than overall survival. CI, confidence interval; *P*_het: *P* value from heterogeneity test.

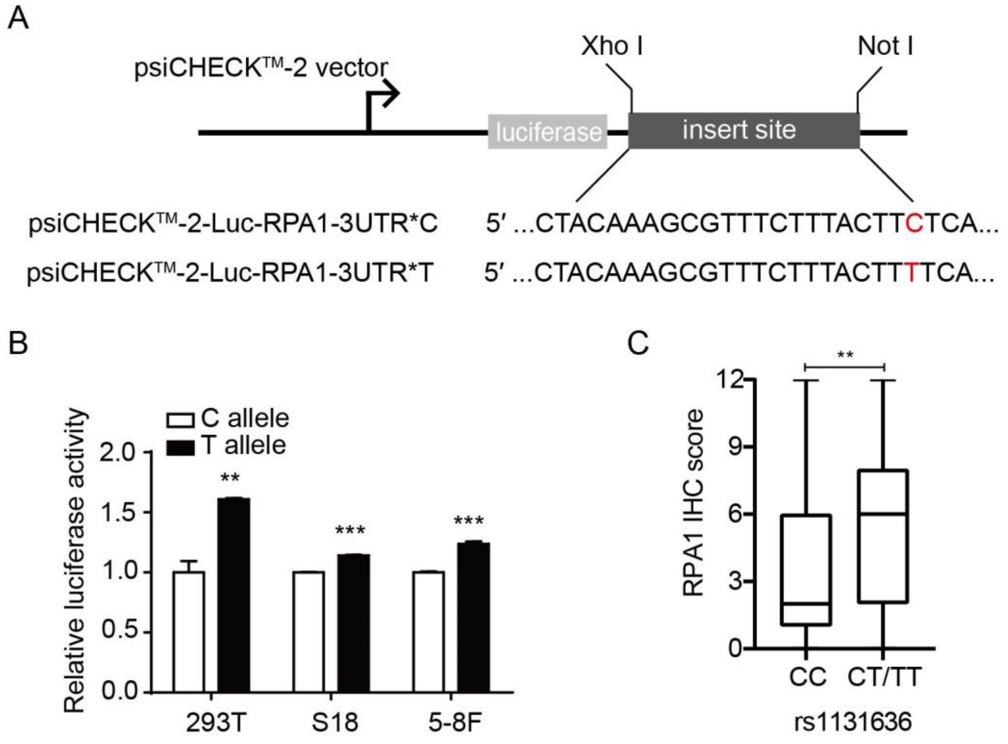

Figure S3. Luciferase reporter assay showing regulatory potential of the variants at rs1131636. (A) Layout of luciferase reporter constructs; the fragment of 3'-UTR of *RPA1* spanning either C or T allele at rs1131636 was inserted downstream of luciferase coding region at psiCHECK2. (B) Luciferase reporter assays were performed in cell lines as indicated, which were transduced with either [C] or [T] constructs as shown above. The Y-axis was relative luciferase activity normalized to that of C allele. Data are shown as mean ± S.D. from three independent experiments. (C) The protein expression of RPA1 in NPC tumour specimens. Box and whisker plot showing the difference in PRA1 protein expression level between the two groups with different genotypes (CC or CT/TT). After the section was immunohistochemistry (IHC) with antibody against RPA1, the expression level of RPA1 was determined based on staining intensity and the percentage of positive cells. Individual genotypes for the matching blood samples were determined using whole-exome sequencing. *P*-value was generated using two-tailed student's *t* test. ***P* < 0.01, ****P* < 0.001.

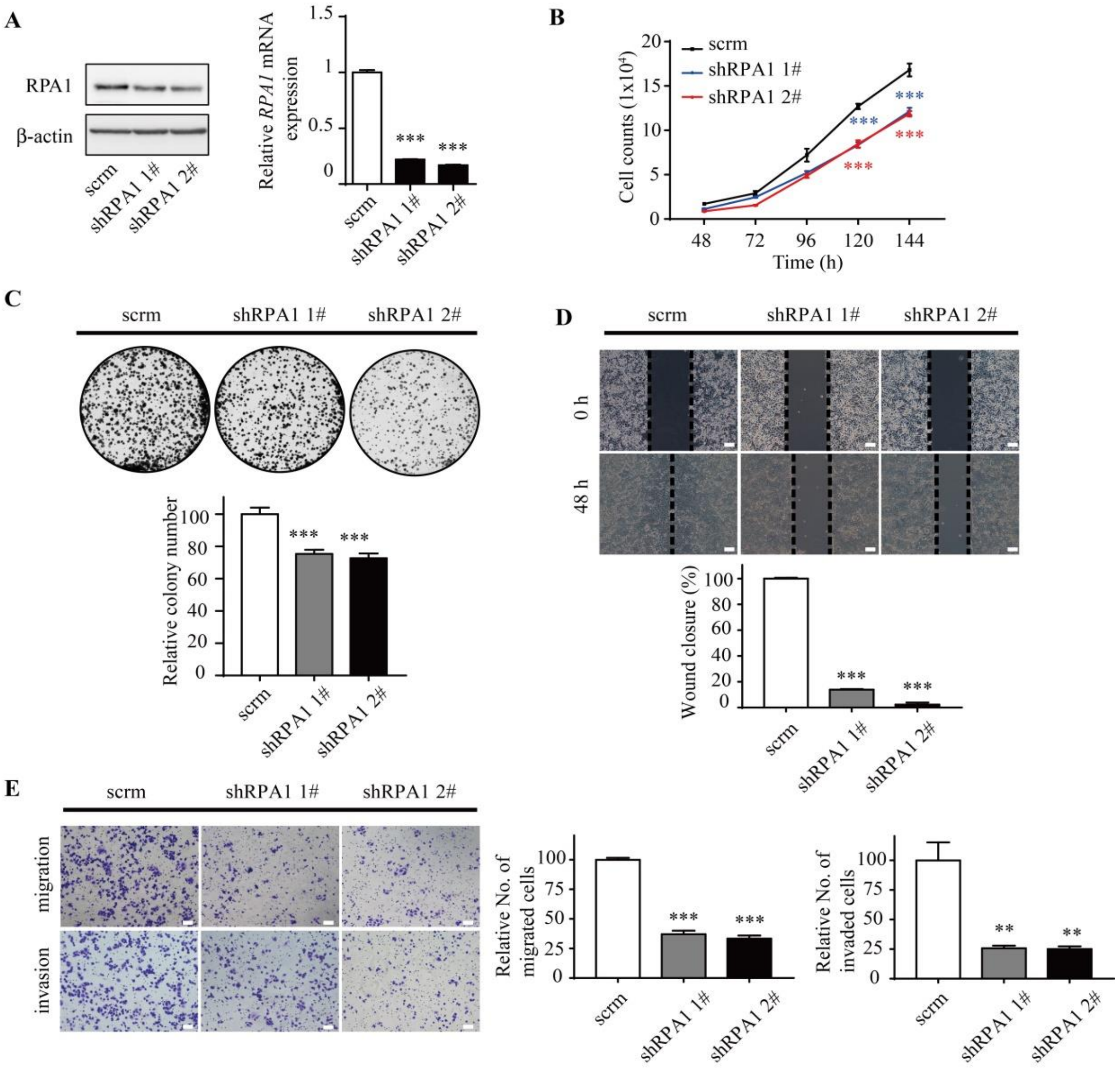

Figure S4. Knockdown of RPA1 inhibited the proliferation, migration, and invasion of 5-8F cells. (A) The expression of RPA1 in 5-8F cells transduced with lentivirus carrying pLKO.1-shRPA1 (shRPA1 1# and shRPA1 2#) was determined by using immunoblotting, as compared to that of pLKO.1-Scrambled-shRNA (scrm). β-actin was served as a loading control. mRNA expression of RPA1 was measured by real-time PCR analysis and normalized to that of β-actin (right panel). (B) Numbers of 5-8F cells transfected with respective lentivirus-construct were determined by averaging the cell numbers in triplicate wells at the indicated time points. (C) Colony forming assay was assessed in 5-8F cells with indicated lentivirus-construct by crystal violet staining method and the representative images were shown at top. Histogram showed the quantification of colony in three independent experiments (bottom). (D) Wound healing assay of 5-8F cells with indicated lentivirus-constructs as being monitored at 0- or 48-hour (h) time-point (top). Scale bar, 100μm. Histogram at bottom represented the relative wound closure rate of three independent experiments. (E) The migration (left top) and invasion (left bottom) abilities of 5-8F cells with indicated lentivirus-constructs were measured by transwell assays without or with Matrigel. Representative images were shown. Scale bar, 100μm. Histograms showed the fold changes relative to the scrambled cells in three independent experiments. All data are shown as mean ± S.D. from at least three independent experiments. *P < 0.05, **P < 0.01, ***P < 0.001.

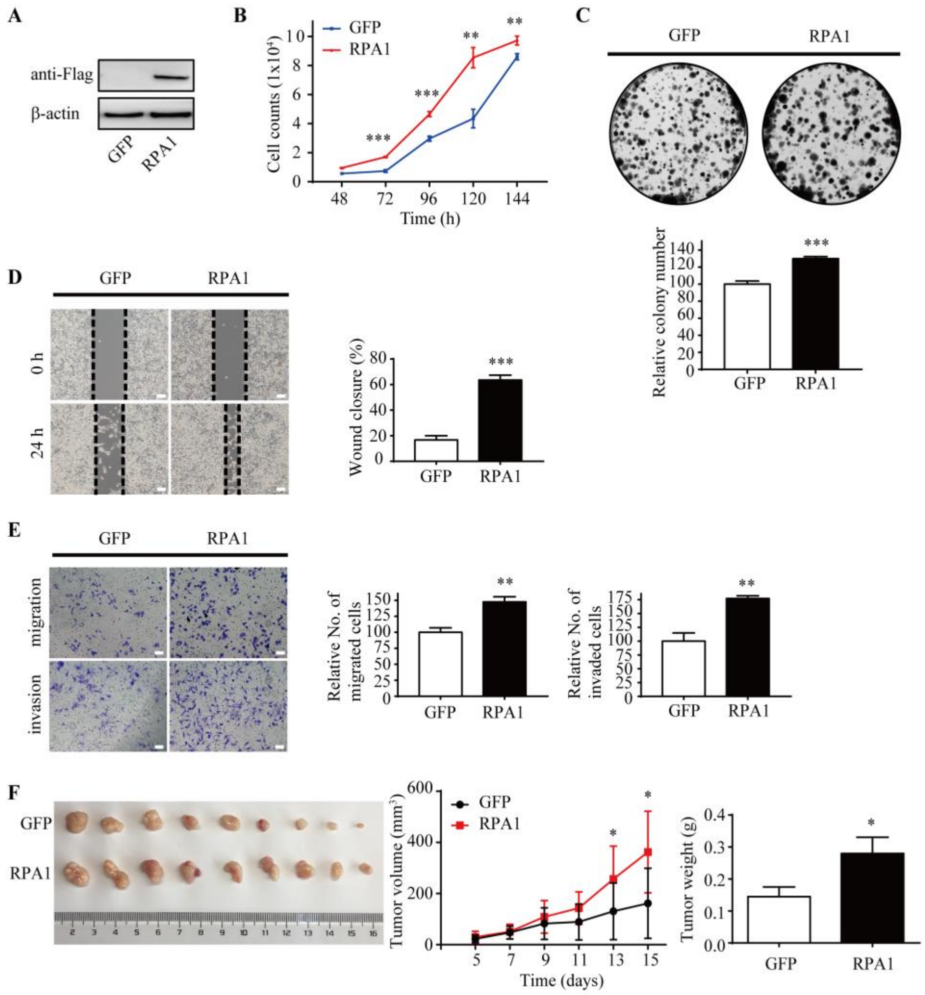

Figure S5. Exogenous expression of RPA1 promoted the proliferation, migration, and invasion of S18 cells. (A) Cell lysates of S18 transduced with lentivirus carrying construct either pCDH-Flag-RPA1 (RPA1) or pCDH-GFP (GFP) as control were subjected to immunoblot with anti-Flag antibody; and β-actin was used as a loading control. (B) Cell proliferation assay showed the number of S18 cells transduced with indicated lentivirus-construct as measured at each time point. (C) Colony formation assay was assessed in cell lines transduced with indicated lentivirus-construct by crystal violet staining method and the representative images were shown at top. Histogram showed the average number of colonies in three independent experiments (bottom). (D) Representative images of wound healing assay in S18 cells transduced with lentivirus-construct as shown. Scale bar, 100μm. Histogram represents the relative wound closure rate of three independent experiments (right). (E) The migration (left top) and invasion (left bottom) abilities of S18 cells with indicated lentivirus-constructs were measured by transwell assays without or with Matrigel. Representative images were shown. Scale bar, 100μm. Histograms showed fold changes relative to pCDH-GFP cells in three independent experiments. (F) Xenograft tumours grown in BALB/c nude mice (n = 9 per group) were shown at left panel, which were subcutaneously injected with S18 cells carrying respective lentivirus-construct as indicated (RPA1 or GFP). The volumes (middle) and the weight (right) of xenograft tumours in nude mice were also measured, respectively. Data are shown as mean ± S.D. from at least three independent experiments. *$P < 0.05$, **$P < 0.01$, ***$P < 0.001$.

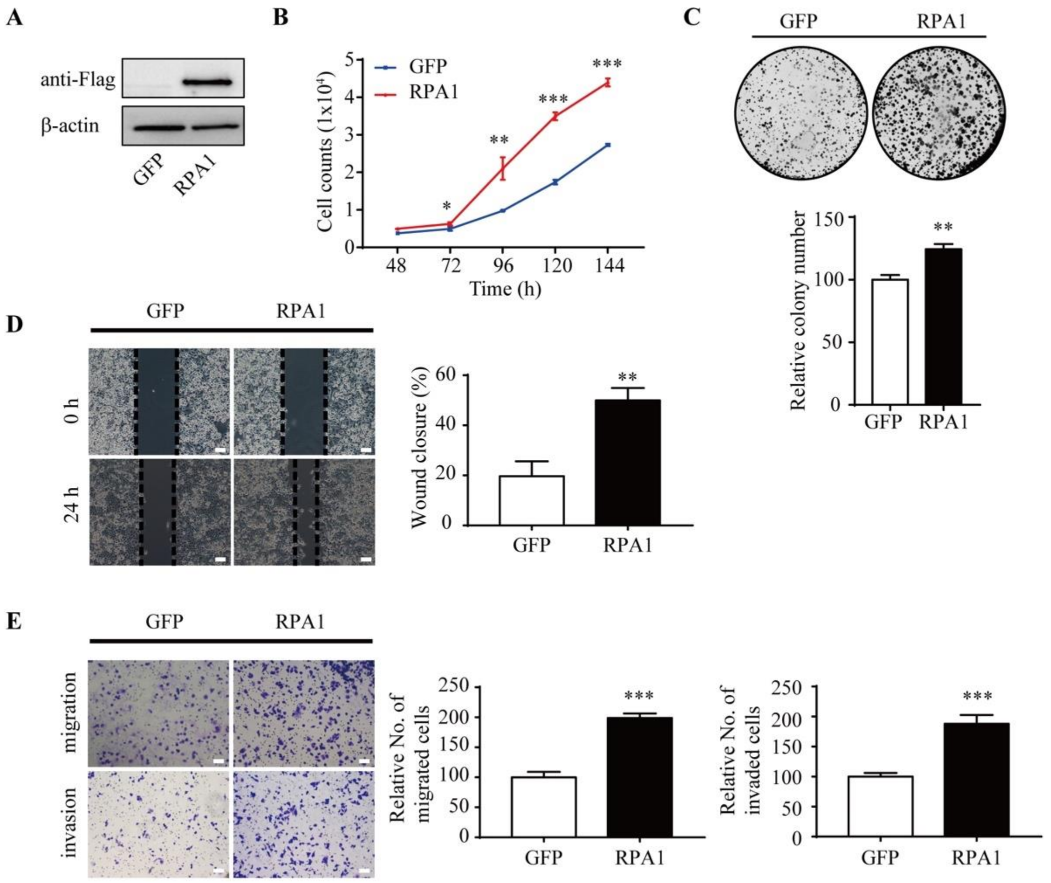

Figure S6. Exogenous expression of RPA1 promoted the proliferation, migration, and invasion of 5-8F cells. (A) Immunoblotting revealed the expression of RPA1 in 5-8F cells transduced with lentivirus carrying pCDH-Flag-RPA1(RPA1) or pCDH-GFP(GFP) as indicated. (B) Cell proliferation assay showed the number of 5-8F cells transduced with indicated lentivirus-construct as measured at each time point. (C) Colony forming assay was assessed in cell lines transduced with indicated lentivirus-construct by crystal violet staining method and the representative images were shown at top. Histogram showed the average number of colonies in three independent experiments (bottom). (D) Representative images of wound healing assay in 5-8F cells transduced with lentivirus-construct as shown. Histogram represented the relative wound closure rate of three independent experiments (right). Scale bar, 100μm. (E) The migration (left top) and invasion (left bottom) abilities of 5-8F cells transduced with lentivirus carrying pCDH-Flag-RPA1 or pCDH-GFP were measured by transwell assays without or with Matrigel. Representative images were shown. Scale bar, 100μm. Histograms showed fold changes relative to the pCDH-GFP cells of three independent experiments. Data are shown as mean ± S.D. from at least three independent experiments. *$P < 0.05$, **$P < 0.01$, ***$P < 0.001$.

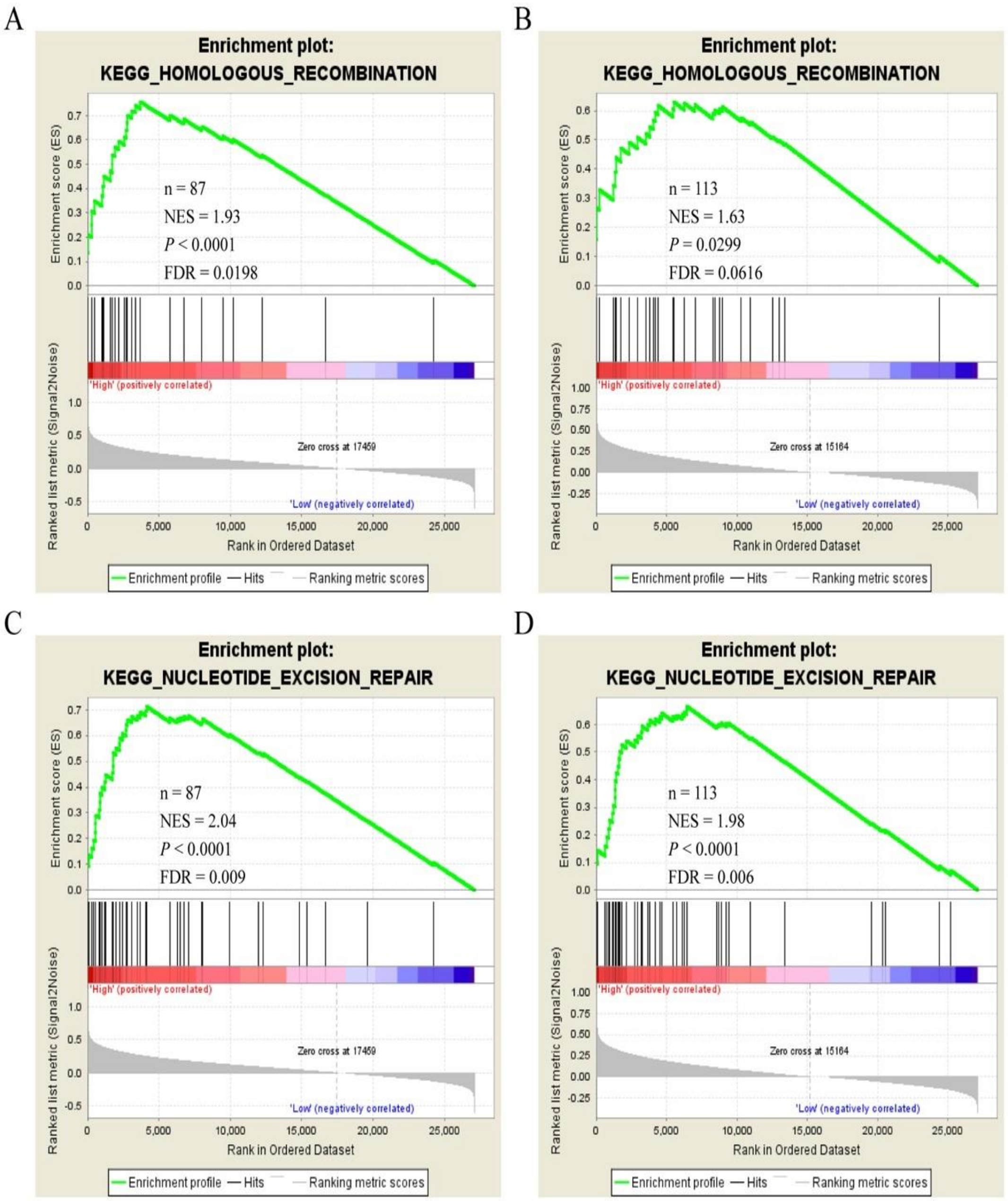

Figure S7. Enrichment plots of results from Gene Set Enrichment Analyses (GSEA). Genes in the homologous recombination pathway were analysed in an in-house mRNA sequencing data (A) and GSE102349 dataset (B), as were genes in the excision repair pathway (C: in-house mRNA sequencing data, D: GSE102349 dataset). ES, normalized enrichment score. Genes are ranked by signal/noise ratio according to their differential expression of RPA1. Genes in the selected gene sets are marked with vertical bars, and the enrichment score is shown as green curve.

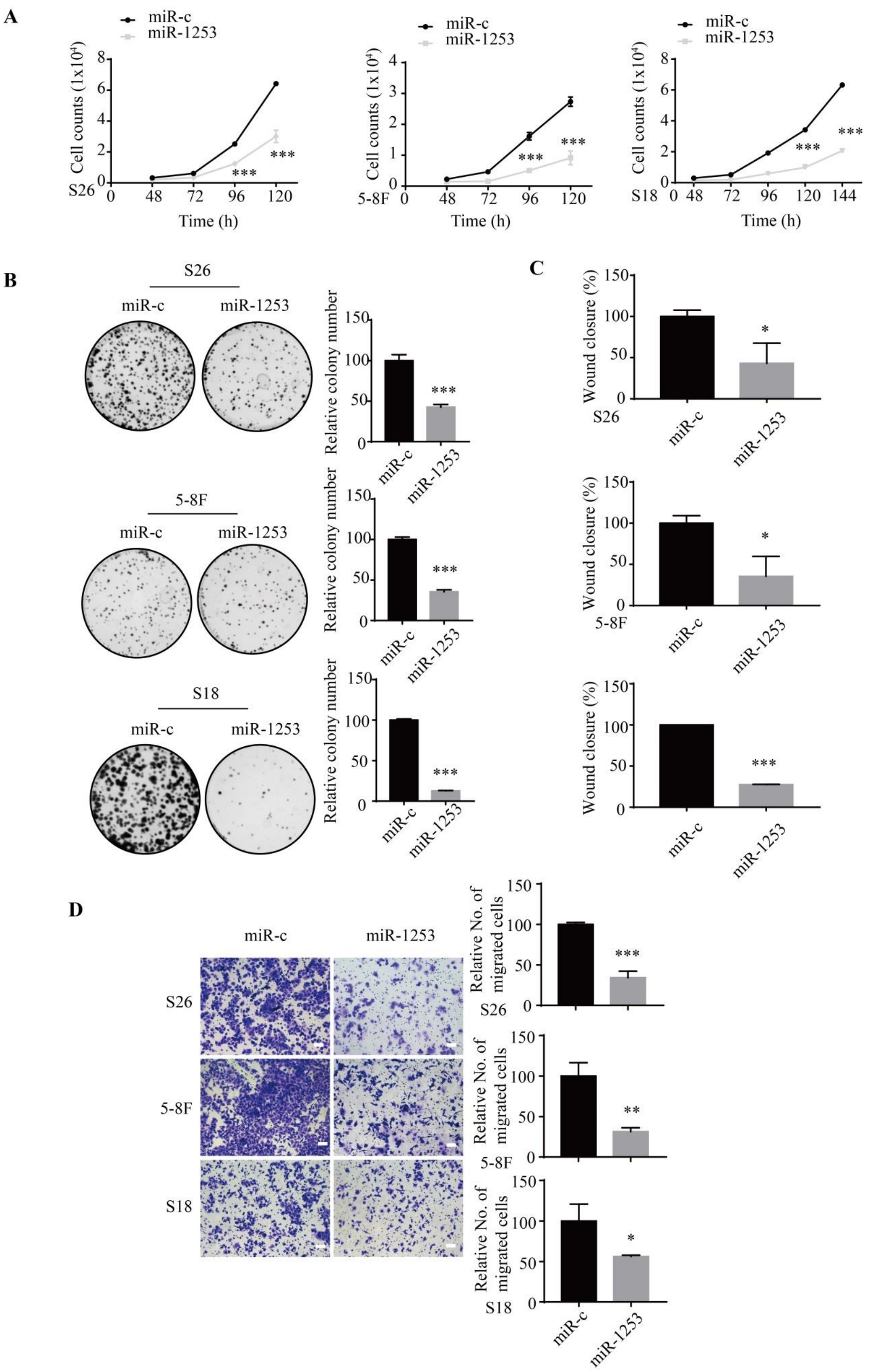

Figure S8. miR-1253 inhibited the proliferation and migration of NPC cells. (A) Numbers of S26, 5-8F and S18 cells transfected with miR-1253 mimics and miR-c were determined by averaging the cell numbers in triplicate wells at the indicated time points. (B) Colony forming assay was assessed in S26, 5-8F and S18 cells by crystal violet staining method and the representative images were shown. (C) Histograms represented the relative wound closure rate of three independent experiments. (D) The migration abilities of S26, 5-8F and S18 cells transfected with miR-1253 were measured and histograms showed the fold changes relative to the scrambled cells in three independent experiments. Scale bar, 100 μm. Data are shown as mean ± S.D. from three independent experiments. *$P < 0.05$, **$P < 0.01$, ***$P < 0.001$.

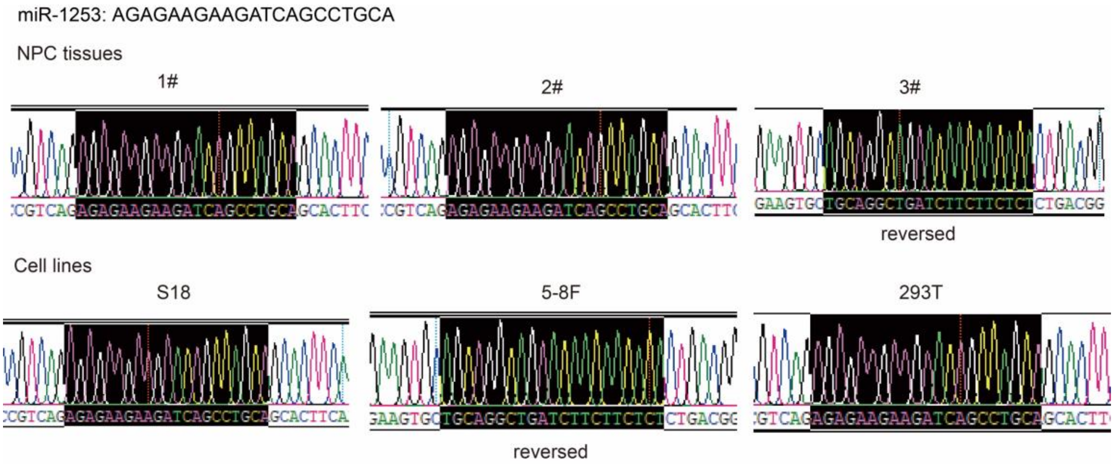

Figure S9. Figure 4. Sequence alignment of miR-1253 from NPC tumours and human cell lines. miR-1253 was amplified and was cloned into pMD™19-T Vector (TAKARA, Japan). Plasmids contained sequences of miR-1253 was determined using Sanger sequencing.

Table S1. Clinical characteristics and overall survival in the discovery and replication cohorts.

Characteristics	Discovery cohorts								Replication cohorts							
	SYSUCC-1				SYSUCC-2				SYSUCC-3				NCCS			
	n	Death (%)	HR (95% CI)	*P*	n	Death (%)	HR (95% CI)	*P*	n	Death (%)	HR (95% CI)	*P*	n	Death (%)	HR (95% CI)	*P*
Total	1,471	346 (23.5)			1,786	256 (14.3)			1751	106 (6.1)			545	69 (12.7)		
Gender																
Male	1,074	283 (26.4)	0.56 (0.42-0.73)	<0.0001		212 (15.8)	0.60 (0.43-0.83)	0.0019	1278	78 (6.1)	0.96 (0.62-1.48)	0.8518	413	62 (15)	0.35 (0.16-0.77)	0.0092
Female	397	63 (15.9)				44 (10)			473	28 (5.9)			132	7 (5.3)		
Age, years																
<50	617	101 (16.4)	1.88 (1.49-2.37)	<0.0001	1,033	117 (11.3)	1.71 (1.33-2.18)	<0.0001	991	45 (4.5)	2.02 (1.38-2.98)	0.0003	249	30 (12)	1.29 (0.80-2.08)	0.2960
>=50	854	245 (28.7)			753	139 (18.5)			760	61 (8)			296	39 (13.2)		
Tumour classification																
T1	198	24 (12.1)			170	12 (7.1)			117	4 (3.4)			162	9 (5.6)		
T2	295	58 (19.7)	1.54 (1.35-1.74)	<0.0001	333	39 (11.7)	1.40 (1.20-1.63)	<0.0001	306	13 (4.2)	1.56 (1.20-2.02)	0.0009	126	20 (15.9)	1.35 (1.08-1.69)	0.0094
T3	700	158 (22.6)			871	124 (14.2)			924	51 (5.5)			156	25 (16)		
T4	278	106 (38.1)			412	81 (19.7)			404	38 (9.4)			101	15 (14.9)		
Lymph node metastasis																
N0	360	56 (15.6)			233	17 (7.3)			228	3 (1.3)			72	7 (9.7)		
N1	446	114 (25.6)	1.36 (1.21-1.54)	<0.0001	725	67 (9.2)	1.90 (1.64-2.21)	<0.0001	702	34 (4.8)	1.87 (1.49-2.36)	<0.0001	182	17 (9.3)	1.38 (1.05-1.83)	0.0216
N2	579	139 (24)			637	114 (17.9)			629	45 (7.2)			214	29 (13.6)		
N3	86	37 (43)			191	58 (30.4)			192	24 (12.5)			77	16 (20.8)		
Distant metastasis																
M0	1,441	333 (23.1)	2.76 (1.58-4.80)	0.0003	1,684	208 (12.4)	6.35 (4.63-8.71)	<0.0001	1712	91 (5.3)	12.7 (7.33-22.0)	<0.0001	540	67 (12.4)	4.70 (1.15-19.2)	0.0314
M1	30	13 (43.3)			102	48 (47.1)			39	15 (38.5)			5	2 (40)		
Clinical stage																
Ⅰ	80	4 (5)			59	0 (0)			44	1 (2.3)			41	3 (7.3)		
Ⅱ	222	34 (15.3)			222	11 (5)			189	3 (1.6)			116	9 (7.8)		
Ⅲ	803	168 (20.9)	1.62 (1.46-1.79)	<0.0001	899	100 (11.1)	1.90 (1.72-2.11)	<0.0001	943	38 (4)	2.23 (1.86-2.67)	<0.0001	225	28 (12.4)	1.41 (1.15-1.75)	0.0012
ⅣA	258	93 (36)			352	59 (16.8)			354	28 (7.9)			83	12 (14.5)		
ⅣB	78	34 (43.6)			152	38 (25)			182	21 (11.5)			75	15 (20)		
ⅣC	30	13 (43.3)			102	48 (47.1)			39	15 (38.5)			5	2 (40)		
IMRT																
No	1,234	295 (23.9)	0.84 (0.63-1.13)	0.2574	747	161 (21.6)	0.44 (0.34-0.56)	<0.0001	0	0 (0)	N/A	N/A	0	0 (0)	N/A	N/A
Yes	237	51 (21.5)			1,039	95 (9.1)			1751	106 (6.1)			545	69 (12.7)		
ICT																
No	850	186 (21.9)	1.26 (1.02-1.56)	0.0311	913	124 (13.6)	1.12 (0.88-1.43)	0.3595	986	56 (5.7)	1.22 (0.83-1.78)	0.3150	475	57 (12)	0.96 (0.51-1.79)	0.8930
Yes	621	160 (25.8)			873	132 (15.1)			765	50 (6.5)			70	12 (17.1)		
CCRT																
No	903	221 (24.5)	0.87 (0.70-1.08)	0.2051	554	91 (16.4)	0.81 (0.62-1.04)	0.0999	261	15 (5.7)	1.09 (0.63-1.88)	0.7536	156	15 (9.6)	1.55 (0.88-2.75)	0.1320
Yes	568	125 (22)			1,232	165 (13.4)			1490	91 (6.1)			389	54 (13.9)		
ACT																
No	1,403	327 (23.3)	1.24 (0.78-1.97)	0.3581	1,736	239 (13.8)	2.67 (1.63-4.37)	<0.0001	1748	104 (5.9)	18.1 (4.46-73.6)	<0.0001	404	52 (12.9)	0.92 (0.54-1.58)	0.7760
Yes	68	19 (27.9)			50	17 (34)			3	2 (66.7)			143	18 (12.6)		
Chemotherapeutic regimens																
RT alone	493	110 (22.3)	Reference		272	43 (15.8)	Reference		165	11 (6.7)	Reference		151	15 (9.9)	Reference	
CCRT alone	309	62 (20.1)	0.89 (0.65-1.22)	0.4642	622	76 (12.2)	0.75 (0.52-1.09)	0.1338	819	44 (5.4)	0.79 (0.41-1.54)	0.4940	181	24 (13.3)	1.91 (1.00-3.66)	0.0512
ICT+RT	399	107 (26.8)	1.15 (1.01-1.32)	0.0349	259	41 (15.8)	0.99 (0.80-1.23)	0.9300	95	4 (4.2)	0.78 (0.44-1.39)	0.4070	5	0 (0)	0.00 (0.00-Inf)	0.9980
CCRT+ACT	42	13 (31)	1.15 (0.95-1.39)	0.1519	16	5 (31.3)	1.25 (0.92-1.70)	0.1600	1	1 (100)	2.63 (1.19-4.70)	0.0144	143	18 (12.6)	1.08 (0.86-1.36)	0.5080
RT+ACT	6	1 (16.7)	0.94 (0.57-1.53)	0.7894	3	0 (0)	0.47 (0.01-16.6)	0.6783	1	0 (0)	0.02 (0.00-Inf)	0.9990	0	0 (0)	N/A	N/A
ICT+RT+ACT	5	3 (60)	1.28 (1.01-1.60)	0.0381	20	7 (35)	1.18 (1.01-1.39)	0.0384	0	0 (0)	N/A	N/A	0	0 (0)	N/A	N/A
ICT+CCRT	202	48 (23.8)	1.01 (0.96-1.07)	0.6277	583	79 (13.6)	0.97 (0.91-1.03)	0.3545	669	45 (6.7)	1.01 (0.91-1.13)	0.8450	65	12 (18.5)	1.05 (0.92-1.19)	0.4510
ICT+CCRT+ACT	15	2 (13.3)	0.92 (0.75-1.12)	0.4156	11	5 (45.5)	1.17 (1.03-1.34)	0.0188	1	1 (100)	1.76 (1.27-2.43)	0.0006	0	0 (0)	N/A	N/A

HR, hazard ratio; CI, confidence interval; RT, radiotherapy; IMRT, intensity modulated radiation therapy; CCRT, concurrent chemoradiotherapy; ICT, induction chemotherapy; ACT, adjuvant chemotherapy. HR and *P* values were derived from univariate Cox proportional hazards regression analyses.

Table S2. Associations results of rs1131636 with overall survival, disease-free survival, distant metastasis-free survival, and local recurrence-free survival in patients with NPC.

Survival	Characteristics	Discovery (SYSUCC-1, SYSUCC-2)				Replication (SYSUCC-3, NCCS)				Meta analysis							
		Univariate		Multivariate		Univariate		Multivariate		Univariate				Multivariate			
		HR (95% CI)	*P*	HR (95% CI)	*P*	HR (95% CI)	*P*	HR (95% CI)	*P*	HR (95% CI)	*P*	I^2	P_{Het}	HR (95% CI)	*P*	I^2	P_{Het}
OS	Gender (Female vs. Male)	0.57 (0.46-0.71)	1.66×10^{-7}	0.63 (0.51-0.77)	1.20×10^{-5}	0.70 (0.48-1.01)	0.059	0.76 (0.52-1.10)	0.1406	0.60 (0.50-0.72)	4.11×10^{-8}	0.00%	0.354	0.65 (0.54-0.79)	5.85×10^{-6}	0.00%	0.384
	Age (>=50 vs. <50 years)	1.77 (1.50-2.09)	1.70×10^{-11}	1.61 (1.36-1.90)	3.24×10^{-8}	1.13 (0.87-1.46)	0.353	1.73 (1.28-2.34)	0.0004	1.76 (1.53-2.04)	1.95×10^{-14}	0.00%	0.938	1.63 (1.41-1.89)	5.34×10^{-11}	0.00%	0.673
	Clinical stage (Advanced vs. Early)	1.74 (1.62-1.87)	2.94×10^{-52}	1.76 (1.63-1.90)	2.12×10^{-50}	1.84 (1.60-2.11)	1.70×10^{-17}	2.00 (1.72-2.32)	5.41×10^{-20}	1.76 (1.65-1.87)	6.00×10^{-68}	0.00%	0.494	1.80 (1.69-1.93)	3.57×10^{-68}	56.2%	0.131
	IMRT (Yes vs. No)	0.62 (0.51-0.75)	7.27×10^{-7}	0.70 (0.57-0.85)	0.0003	N/A	N/A	N/A	N/A	N/A	N/A	N/A	N/A	N/A	N/A	N/A	N/A
	ICT (Yes vs. No)	1.20 (1.02-1.41)	0.0254	0.79 (0.66-0.93)	0.0054	1.05 (0.77-1.44)	0.7418	0.76 (0.54-1.06)	0.1036	1.17 (1.01-1.35)	0.0325	0.00%	0.466	0.78 (0.67-0.91)	0.0013	0.00%	0.849
	CCRT (Yes vs. No)	0.86 (0.73-1.01)	0.0582	0.81 (0.69-0.96)	0.0172	1.24 (0.84-1.84)	0.2849	0.64 (0.42-0.98)	0.0411	0.90 (0.78-1.05)	0.1762	65.5%	0.088	0.79 (0.67-0.92)	0.0030	1.18%	0.314
	ACT (Yes vs. No)	1.68 (1.20-2.35)	0.0027	1.52 (1.08-2.14)	0.0175	1.29 (0.80-2.08)	0.3045	0.95 (0.57-1.59)	0.8582	1.54 (1.17-2.02)	0.0023	0.00%	0.377	1.31 (0.99-1.75)	0.0614	54.1%	0.140
	rs1131636 (Additive model)	1.38 (1.23-1.55)	6.28×10^{-8}	1.35 (1.20-1.51)	7.21×10^{-7}	1.22 (0.98-1.51)	0.0741	1.27 (1.03-1.58)	0.0269	1.34 (1.21-1.49)	2.07×10^{-8}	3.57%	0.309	1.33 (1.20-1.47)	6.31×10^{-8}	0.00%	0.970
DFS	Gender (Female vs. Male)	0.64 (0.54-0.77)	9.25×10^{-7}	0.69 (0.58-0.82)	3.60×10^{-5}	0.74 (0.59-0.95)	0.0153	0.77 (0.60-0.98)	0.0314	0.68 (0.59-0.78)	7.07×10^{-8}	0.00%	0.340	0.71 (0.62-0.82)	4.15×10^{-6}	0.00%	0.467
	Age (>=50 vs. <50 years)	1.40 (1.21-1.61)	3.00×10^{-6}	1.26 (1.09-1.45)	0.0015	1.26 (1.03-1.53)	0.0221	1.22 (1.00-1.49)	0.0503	1.35 (1.20-1.51)	3.29×10^{-7}	0.00%	0.406	1.25 (1.11-1.40)	0.0002	0.00%	0.797
	Clinical stage (Advanced vs. Early)	1.54 (1.45-1.64)	2.30×10^{-40}	1.57 (1.47-1.68)	1.60×10^{-41}	1.53 (1.39-1.68)	4.30×10^{-18}	1.64 (1.48-1.82)	4.44×10^{-21}	1.54 (1.46-1.62)	9.23×10^{-57}	0.00%	0.884	1.59 (1.50-1.68)	8.70×10^{-61}	0.00%	0.505
	IMRT (Yes vs. No)	0.61 (0.52-0.71)	6.00×10^{-10}	0.67 (0.57-0.79)	3.00×10^{-6}	N/A	N/A	N/A	N/A	N/A	N/A	N/A	N/A	N/A	N/A	N/A	N/A
	ICT (Yes vs. No)	1.11 (0.97-1.28)	0.1363	0.81 (0.70-0.94)	0.0043	1.16 (0.95-1.42)	0.1532	0.90 (0.72-1.12)	0.3472	1.13 (1.00-1.26)	0.0416	0.00%	0.741	0.83 (0.74-0.94)	0.0038	0.00%	0.418
	CCRT (Yes vs. No)	0.80 (0.69-0.92)	0.0015	0.78 (0.68-0.91)	0.0014	0.95 (0.74-1.23)	0.7164	0.59 (0.45-0.77)	0.0001	0.83 (0.74-0.94)	0.0031	33.1%	0.221	0.73 (0.64-0.84)	3.35×10^{-6}	70.1%	0.068
	ACT (Yes vs. No)	1.57 (1.15-2.14)	0.0047	1.43 (1.04-1.96)	0.027	1.23 (0.86-1.77)	0.255	1.10 (0.75-1.61)	0.6194	1.41 (1.12-1.79)	0.0039	0.00%	0.328	1.28 (1.01-1.64)	0.0436	6.14%	0.302
	rs1131636 (Additive model)	1.26 (1.14-1.39)	1.00×10^{-5}	1.23 (1.11-1.36)	5.09×10^{-5}	1.02 (0.88-1.17)	0.8379	1.04 (0.90-1.20)	0.604	1.17 (1.08-1.27)	0.0002	82.7%	0.016	1.16 (1.07-1.26)	0.0003	20.4%	0.290
DMFS	Gender (Female vs. Male)	0.62 (0.51-0.76)	5.00×10^{-6}	0.69 (0.56-0.84)	0.0003	0.71 (0.52-0.97)	0.0329	0.73 (0.53-1.01)	0.054	0.65 (0.54-0.77)	5.42×10^{-7}	0.00%	0.504	0.70 (0.59-0.83)	4.89×10^{-5}	0.00%	0.750
	Age (>=50 vs. <50 years)	1.54 (1.31-1.81)	1.60×10^{-7}	1.38 (1.17-1.63)	0.0001	0.98 (0.76-1.27)	0.8671	0.96 (0.74-1.25)	0.7549	1.36 (1.18-1.55)	1.29×10^{-5}	88.2%	0.004	1.25 (1.09-1.43)	0.0017	81.5%	0.020
	Clinical stage (Advanced vs. Early)	1.71 (1.60-1.84)	6.58×10^{-51}	1.73 (1.61-1.86)	2.27×10^{-50}	1.57 (1.39-1.78)	8.49×10^{-13}	1.61 (1.41-1.85)	3.08×10^{-12}	1.68 (1.58-1.78)	9.55×10^{-62}	30.8%	0.229	1.71 (1.60-1.82)	8.67×10^{-61}	0.00%	0.355
	IMRT (Yes vs. No)	0.55 (0.46-0.66)	1.92×10^{-10}	0.61 (0.51-0.74)	6.19×10^{-7}	N/A	N/A	N/A	N/A	N/A	N/A	N/A	N/A	N/A	N/A	N/A	N/A
	ICT (Yes vs. No)	1.16 (0.99-1.35)	0.0726	0.80 (0.67-0.94)	0.0074	1.20 (0.93-1.56)	0.1654	0.98 (0.73-1.31)	0.8835	1.17 (1.02-1.34)	0.0242	0.00%	0.795	0.84 (0.73-0.97)	0.0167	31.9%	0.226
	CCRT (Yes vs. No)	0.83 (0.71-0.97)	0.0188	0.82 (0.70-0.97)	0.0221	1.20 (0.84-1.70)	0.3182	0.69 (0.47-1.01)	0.0535	0.88 (0.76-1.02)	0.0832	71.5%	0.061	0.80 (0.69-0.93)	0.0040	0.00%	0.403
	ACT (Yes vs. No)	1.85 (1.33-2.57)	0.0002	1.63 (1.16-2.27)	0.0044	1.83 (1.21-2.76)	0.004	1.65 (1.07-2.57)	0.0247	1.84 (1.42-2.38)	3.17×10^{-6}	0.00%	0.966	1.64 (1.25-2.13)	0.0003	0.00%	0.950
	rs1131636 (Additive model)	1.26 (1.13-1.42)	6.40×10^{-5}	1.23 (1.10-1.38)	0.0004	0.97 (0.81-1.17)	0.7717	0.99 (0.82-1.19)	0.891	1.17 (1.07-1.29)	0.0012	82.2%	0.018	1.16 (1.05-1.28)	0.0033	39.4%	0.180
LRFS	Gender (Female vs. Male)	0.72 (0.52-0.98)	0.0362	0.72 (0.53-0.99)	0.0436	0.73 (0.48-1.11)	0.1432	0.74 (0.48-1.13)	0.1655	0.72 (0.56-0.93)	0.0106	0.00%	0.951	0.73 (0.57-0.94)	0.0144	0.00%	0.935
	Age (>=50 vs. <50 years)	1.13 (0.88-1.46)	0.3535	1.06 (0.82-1.37)	0.6583	1.34 (0.95-1.90)	0.0952	1.25 (0.89-1.78)	0.2035	1.20 (0.98-1.47)	0.0824	0.00%	0.428	1.12 (0.91-1.38)	0.2660	0.00%	0.448
	Clinical stage (Advanced vs. Early)	1.09 (0.96-1.24)	0.1706	1.10 (0.96-1.25)	0.1837	1.11 (0.93-1.32)	0.2504	1.32 (1.09-1.59)	0.0039	1.10 (0.99-1.22)	0.0744	0.00%	0.902	1.17 (1.05-1.30)	0.0058	59.4%	0.116
	IMRT (Yes vs. No)	0.80 (0.61-1.06)	0.1138	0.87 (0.65-1.17)	0.3551	N/A	N/A	N/A	N/A	N/A	N/A	N/A	N/A	N/A	N/A	N/A	N/A
	ICT (Yes vs. No)	1.14 (0.89-1.47)	0.3059	1.04 (0.79-1.37)	0.7712	0.79 (0.54-1.15)	0.209	0.74 (0.49-1.11)	0.1404	1.02 (0.82-1.25)	0.8843	61.6%	0.107	0.93 (0.74-1.17)	0.5626	48.0%	0.166
	CCRT (Yes vs. No)	0.75 (0.58-0.97)	0.0269	0.76 (0.57-0.99)	0.0464	0.59 (0.40-0.87)	0.0076	0.45 (0.29-0.70)	0.0004	0.70 (0.56-0.86)	0.0009	1.16%	0.314	0.65 (0.52-0.82)	0.0003	74.1%	0.050
	ACT (Yes vs. No)	1.07 (0.55-2.09)	0.8373	1.10 (0.56-2.15)	0.7893	1.21 (0.66-2.23)	0.5449	1.17 (0.61-2.23)	0.6358	1.14 (0.73-1.79)	0.5585	0.00%	0.796	1.13 (0.71-1.80)	0.5983	0.00%	0.893
	rs1131636 (Additive model)	1.20 (0.99-1.44)	0.0529	1.20 (1.00-1.44)	0.0561	1.16 (0.91-1.49)	0.24	1.14 (0.89-1.46)	0.311	1.19 (1.02-1.37)	0.0241	0.00%	0.838	1.17 (1.01-1.36)	0.0325	0.00%	0.920

HR, hazard ratio; CI, confidence interval; OS, overall survival; DFS, disease-free survival; DMFS, distant metastasis-free survival; LRFS, localregional recurrence-free survival. HR and *P* values for discovery and replication cohorts were derived from the Cox proportional hazards regression analyses; Meta-analysis was performed under fixed-effect model.

Table S3. Distribution of rs1131636 genotypes stratified by clinical characteristics in the discovery cohorts, validation cohorts and combined samples.

Characteristics	Discovery cohorts										Replication cohorts										Combined samples				
	SYSUCC-1					SYSUCC-2					SYSUCC-3					NCCS									
	n	rs1131636, n (%)			*P*	n	rs1131636, n (%)			*P*	n	rs1131636, n (%)			*P*	n	rs1131636, n (%)			*P*	n	rs1131636, n (%)			*P*
		CC	CT	TT			CC	CT	TT			CC	CT	TT			CC	CT	TT			CC	CT	TT	
Total	1471	293 (19.9)	741 (50.4)	437 (29.7)		1786	364 (20.4)	914 (51.2)	508 (28.4)		1751	356 (20.3)	882 (50.4)	513 (29.3)		545	101 (18.5)	264 (48.4)	180 (33.0)		5553	1114 (20.1)	2801 (50.4)	1638 (29.5)	
Gender																									
Male	1074	212 (19.7)	539 (50.2)	323 (30.1)	0.8728	1345	262 (19.5)	688 (51.2)	395 (29.4)	0.149	1278	251 (19.6)	645 (50.5)	382 (29.9)	0.4299	413	78 (18.9)	197 (47.7)	138 (33.4)	0.8247	4110	803 (19.5)	2069 (50.3)	1238 (30.1)	0.1176
Female	397	81 (20.4)	202 (50.9)	114 (28.7)		441	102 (23.1)	226 (51.2)	113 (25.6)		473	105 (22.2)	237 (50.1)	131 (27.7)		132	23 (17.4)	67 (50.8)	42 (31.8)		1443	311 (21.6)	732 (50.7)	400 (27.7)	
Age, years																									
<50	617	122 (19.8)	317 (51.4)	178 (28.8)	0.7837	1033	219 (21.2)	518 (50.1)	296 (28.7)	0.508	991	202 (20.4)	507 (51.2)	282 (28.5)	0.658	249	48 (19.3)	122 (49)	79 (31.7)	0.8182	2890	591 (20.4)	1464 (50.7)	835 (28.9)	0.5334
>=50	854	171 (20)	424 (49.6)	259 (30.3)		753	145 (19.3)	396 (52.6)	212 (28.2)		760	154 (20.3)	375 (49.3)	231 (30.4)		296	53 (17.9)	142 (48)	101 (34.1)		2663	523 (19.6)	1337 (50.2)	803 (30.2)	
Tumour classification																									
T1	198	46 (23.2)	108 (54.5)	44 (22.2)		170	33 (19.4)	87 (51.2)	50 (29.4)		117	24 (20.5)	54 (46.2)	39 (33.3)		162	30 (18.5)	77 (47.5)	55 (34)		647	133 (20.6)	326 (50.4)	188 (29.1)	
T2	295	51 (17.3)	150 (50.8)	94 (31.9)	0.1842	333	68 (20.4)	175 (52.6)	90 (27)	0.2315	306	69 (22.5)	148 (48.4)	89 (29.1)	0.7236	126	26 (20.6)	57 (45.2)	43 (34.1)	0.5856	1060	214 (20.2)	530 (50)	316 (29.8)	0.2451
T3	700	140 (20)	353 (50.4)	207 (29.6)		871	195 (22.4)	441 (50.6)	235 (27)		924	183 (19.8)	481 (52.1)	260 (28.1)		156	33 (21.2)	76 (48.7)	47 (30.1)		2651	551 (20.8)	1351 (51)	749 (28.3)	
T4	278	56 (20.1)	130 (46.8)	92 (33.1)		412	68 (16.5)	211 (51.2)	133 (32.3)		404	80 (19.8)	199 (49.3)	125 (30.9)		101	12 (11.9)	54 (53.5)	35 (34.7)		1195	216 (18.1)	594 (49.7)	385 (32.2)	
Lymph node metastasis																									
N0	360	69 (19.2)	192 (53.3)	99 (27.5)		233	37 (15.9)	125 (53.6)	71 (30.5)		228	45 (19.7)	110 (48.2)	73 (32.1)		72	15 (20.8)	34 (47.2)	23 (31.9)		893	166 (18.6)	461 (51.6)	266 (29.8)	
N1	446	95 (21.3)	227 (50.9)	124 (27.8)	0.0949	725	167 (23)	364 (50.2)	194 (26.8)	0.2876	702	142 (20.2)	353 (50.3)	207 (29.5)	0.9071	182	37 (20.3)	80 (44)	65 (35.7)	0.7522	2055	441 (21.5)	1024 (49.8)	590 (28.7)	0.4151
N2	579	106 (18.3)	291 (50.3)	182 (31.4)		637	126 (19.8)	326 (51.2)	185 (29)		629	125 (19.9)	322 (51.2)	182 (28.9)		214	34 (15.9)	111 (51.9)	69 (32.2)		2059	391 (19)	1050 (51)	618 (30)	
N3	86	23 (26.7)	31 (36)	32 (37.2)		191	34 (17.8)	99 (51.8)	58 (30.4)		192	44 (22.9)	97 (50.5)	51 (26.6)		77	15 (19.5)	39 (50.6)	23 (29.9)		546	116 (21.2)	266 (48.7)	164 (30.1)	
Distant metastasis																									
M0	1441	288 (20)	725 (50.3)	428 (29.7)	0.8973	1684	355 (21.1)	858 (51)	471 (28)	0.0078	1712	345 (20.2)	861 (50.3)	506 (29.5)	0.2159	540	99 (18.3)	263 (48.7)	178 (33)	0.3376	5377	1087 (20.2)	2707 (50.3)	1583 (29.5)	0.2829
M1	30	5 (16.7)	16 (53.3)	9 (30)		102	9 (8.8)	56 (54.9)	37 (36.3)		39	11 (28.3)	21 (53.8)	7 (17.9)		5	2 (40)	1 (20)	2 (40)		176	27 (15.3)	94 (53.4)	55 (31.3)	
Clinical stage																									
Ⅰ	80	19 (23.8)	41 (51.3)	20 (25)		59	7 (11.9)	31 (52.5)	21 (35.6)		44	9 (20.5)	17 (38.6)	18 (40.9)		41	10 (24.4)	21 (51.2)	10 (24.4)		224	45 (20.1)	110 (49.1)	69 (30.8)	
Ⅱ	222	43 (19.4)	120 (54.1)	59 (26.6)		222	43 (19.4)	118 (53.2)	61 (27.5)		189	45 (23.8)	87 (46)	57 (30.2)		116	22 (19)	51 (44)	43 (37.1)		749	153 (20.4)	376 (50.2)	220 (29.4)	
Ⅲ	803	153 (19.1)	417 (51.9)	233 (29)	0.1645	899	211 (23.5)	457 (50.8)	231 (25.7)	0.0093	943	182 (19.3)	489 (51.9)	272 (28.8)	0.3345	225	43 (19.1)	110 (48.9)	72 (32)	0.626	2870	589 (20.5)	1473 (51.3)	808 (28.2)	0.2386
ⅣA	258	52 (20.2)	121 (46.9)	85 (32.9)		352	62 (17.6)	174 (49.4)	116 (33)		354	68 (19.2)	174 (49.2)	112 (31.6)		83	10 (12)	42 (50.6)	31 (37.4)		1047	192 (18.3)	511 (48.8)	344 (32.9)	
ⅣB	78	21 (26.9)	26 (33.3)	31 (39.7)		152	32 (21.1)	78 (51.3)	42 (27.6)		182	41 (22.5)	94 (51.6)	47 (25.9)		75	14 (18.7)	39 (52)	22 (29.3)		487	108 (22.2)	237 (48.7)	142 (29.1)	
ⅣC	30	5 (16.7)	16 (53.3)	9 (30)		102	9 (8.8)	56 (54.9)	37 (36.3)		39	11 (28.2)	21 (53.8)	7 (18)		5	2 (40)	1 (20)	2 (40)		176	27 (15.3)	94 (53.4)	55 (31.3)	
IMRT																									
No	1234	243 (19.7)	623 (50.5)	368 (29.8)	0.8826	747	145 (19.4)	390 (52.2)	212 (28.4)	0.6498	0	0 (0)	0 (0)	0 (0)	N/A	0	0 (0)	0 (0)	0 (0)	N/A	1981	388 (19.6)	1013 (51.1)	580 (29.3)	0.7077
Yes	237	50 (21.1)	118 (49.8)	69 (29.1)		1039	219 (21.1)	524 (50.4)	296 (28.5)		1751	356 (20.3)	882 (50.4)	513 (29.3)		545	101 (18.5)	264 (48.4)	180 (33)		3572	726 (20.3)	1788 (50.1)	1058 (29.6)	
ICT																									
No	850	169 (19.9)	437 (51.4)	244 (28.7)	0.5735	913	191 (20.9)	471 (51.6)	251 (27.5)	0.6302	986	200 (20.3)	484 (49.1)	302 (30.6)	0.3488	475	91 (19.2)	229 (48.2)	155 (32.6)	0.6073	3224	651 (20.2)	1621 (50.3)	952 (29.5)	0.9473
Yes	621	124 (20)	304 (49)	193 (31.1)		873	173 (19.8)	443 (50.7)	257 (29.4)		765	156 (20.4)	398 (52)	211 (27.8)		70	10 (14.3)	35 (50)	25 (35.7)		2329	463 (19.9)	1180 (50.7)	686 (29.4)	
CCRT																									
No	903	177 (19.6)	458 (50.7)	268 (29.7)	0.9166	554	100 (18.1)	293 (52.9)	161 (29.1)	0.2583	261	61 (23.4)	136 (52.1)	64 (24.5)	0.1376	156	37 (23.7)	68 (43.6)	51 (32.7)	0.1199	1874	375 (20)	955 (51)	544 (29)	0.833
Yes	568	116 (20.4)	283 (49.8)	169 (29.8)		1232	264 (21.4)	621 (50.4)	347 (28.2)		1490	295 (19.8)	746 (50.1)	449 (30.1)		389	64 (16.5)	196 (50.4)	129 (33.2)		3679	739 (20.1)	1846 (50.2)	1094 (29.7)	
ACT																									
No	1403	277 (19.7)	706 (50.3)	420 (29.9)	0.6018	1736	352 (20.3)	891 (51.3)	493 (28.4)	0.7248	1748	355 (20.3)	881 (50.4)	512 (29.3)	0.8026	402	76 (18.9)	191 (47.5)	135 (33.6)	0.7671	5289	1060 (20)	2669 (50.5)	1560 (29.5)	0.984
Yes	68	16 (23.5)	35 (51.5)	17 (25)		50	12 (24)	23 (46)	15 (30)		3	1 (33.3)	1 (33.3)	1 (33.3)		143	25 (17.5)	73 (51)	45 (31.5)		264	54 (20.5)	132 (50)	78 (29.5)	

IMRT, intensity modulated radiation therapy;CCRT, concurrent chemoradiotherapy; ICT, induction chemotherapy; ACT, adjuvant chemotherapy. *P* values were derived from Pearson's Chi-squared test.

Table S4. Allele frequencies of rs1131636 in NPC cohorts and general populations from 1000 Genomes Project (https://www.internationalgenome.org/).

Population	n	Allele frequency		rs1131636, n (%)		
		C	T	CC	CT	TT
NPC cohorts						
ALL	5553	0.453	0.547	1114 (20.1)	2801 (50.4)	1638 (29.5)
SYSUCC-1	1471	0.451	0.549	293 (19.9)	741 (50.4)	437 (29.7)
SYSUCC-2	1786	0.46	0.54	364 (20.4)	914 (51.2)	508 (28.4)
SYSUCC-3	1751	0.455	0.545	356 (20.3)	882 (50.4)	513 (29.3)
NCCS	545	0.428	0.572	101 (18.5)	264 (48.4)	180 (33.0)
1000 Genomes Project (Phase 3)						
ALL	2504	0.535	0.465	753 (30.1)	1175 (46.9)	576 (23)
African	661	0.643	0.357	278 (42.1)	294 (44.5)	89 (13.5)
African Caribbeans in Barbados	96	0.625	0.375	36 (37.5)	48 (50)	12 (12.5)
Americans of African Ancestry in SW USA	61	0.615	0.385	23 (37.7)	29 (47.5)	9 (14.8)
Esan in Nigeria	99	0.672	0.328	42 (42.4)	49 (49.5)	8 (8.1)
Gambian in Western Divisions in the Gambia	113	0.571	0.429	38 (33.6)	53 (46.9)	22 (19.5)
Luhya in Webuye, Kenya	99	0.672	0.328	46 (46.5)	41 (41.4)	12 (12.1)
Mende in Sierra Leone	85	0.676	0.324	40 (47.1)	35 (41.2)	10 (11.8)
Yoruba in Ibadan, Nigeria	108	0.671	0.329	53 (49.1)	39 (36.1)	16 (14.8)
Ad Mixed American	347	0.465	0.535	75 (21.6)	173 (49.9)	99 (28.5)
Colombians from Medellin, Colombia	94	0.5	0.5	21 (22.3)	52 (55.3)	21 (22.3)
Mexican Ancestry from Los Angeles USA	64	0.406	0.594	11 (17.2)	30 (46.9)	23 (35.9)
Peruvians from Lima, Peru	85	0.476	0.524	21 (24.7)	39 (45.9)	25 (29.4)
Puerto Ricans from Puerto Rico	104	0.462	0.538	22 (21.2)	52 (50)	30 (28.8)
East Asian	504	0.476	0.524	115 (22.8)	250 (49.6)	139 (27.6)
Chinese Dai in Xishuangbanna, China	93	0.484	0.516	18 (19.4)	54 (58.1)	21 (22.6)
Han Chinese in Beijing, China	103	0.447	0.553	23 (22.3)	46 (44.7)	34 (33)
Southern Han Chinese	105	0.419	0.581	19 (18.1)	50 (47.6)	36 (34.3)
Japanese in Tokyo, Japan	104	0.476	0.524	25 (24)	49 (47.1)	30 (28.8)
Kinh in Ho Chi Minh City, Vietnam	99	0.561	0.439	30 (30.3)	51 (51.5)	18 (18.2)
European	503	0.374	0.626	66 (13.1)	244 (48.5)	193 (38.4)
Utah Residents (CEPH) with Northern and Western European Ancestry	99	0.348	0.652	13 (13.1)	43 (43.4)	43 (43.4)
Finnish in Finland	99	0.348	0.652	9 (9.1)	51 (51.5)	39 (39.4)
British in England and Scotland	91	0.374	0.626	12 (13.2)	44 (48.4)	35 (38.5)
Iberian Population in Spain	107	0.421	0.579	18 (16.8)	54 (50.5)	35 (32.7)
Toscani in Italia	107	0.374	0.626	14 (13.1)	52 (48.6)	41 (38.3)
South Asian	489	0.667	0.333	219 (44.8)	214 (43.8)	56 (11.5)
Bengali from Bangladesh	86	0.663	0.337	39 (45.3)	36 (41.9)	11 (12.8)
Gujarati Indian from Houston, Texas	103	0.684	0.316	47 (45.6)	47 (45.6)	9 (8.7)
Indian Telugu from the UK	102	0.632	0.368	44 (43.1)	41 (40.2)	17 (16.7)
Punjabi from Lahore, Pakistan	96	0.63	0.37	36 (37.5)	49 (51)	11 (11.5)
Sri Lankan Tamil from the UK	102	0.721	0.279	53 (52)	41 (40.2)	8 (7.8)

Table S5. ***Cis*** **-eQTL effects of rs1131636 on RPA1 expression from GTEx portal.**

SNP	Chromosome	Position	Gene Symbol	Normalized effect size	*P* -value	Tissue
rs1131636	17	1801189	RPA1	-0.23	3.6×10^{-9}	Tibial nerve
				-0.16	4.1×10^{-7}	Skeletal muscle
				-0.35	4.1×10^{-6}	Brain cortex
				-0.24	1.0×10^{-5}	Adrenal gland
				-0.1	2.5×10^{-5}	Oesophagus mucosa

* https://gtexportal.org/home/

Table S6. ***Cis*** **-eQTL effects of rs1131636 on RPA1 expression from Blood eQTL browser.**

SNP	Chromosome	Position	Gene Symbol	Z-score	*P* -value	Tissue
rs1131636	17	1801189	RPA1	-3.610	0.0003	Peripheral blood

* https://genenetwork.nl/bloodeqtlbrowser/

Table S7. Sequences of oligonucleotides used in this study.

Experiment	Oligo name	Sequence
Primers for qPCR	RPA1-q-F	5'-AAGGAGCCCGAGTCTCTGAT-3'
	RPA1-q-R	5'-TGGTGTTACTCCCTCCGACT-3'
	β-actin-q-F	5'-CCCACACTGTGCCCATCTAC-3'
	β-actin-q-R	5'-GGAACCGCTCATTGCCAATG-3'
Primers for cloning	RPA1-3'UTR-F	5'-GAGGAGCAGTGCCAATCGGGC-3'
	RPA1-3'UTR-R	5'-AGAGATTAGCAAGGTTTTAAT-3'
	Flag-RPA1-FL-F	5'-ATGGACTACAAAGACGATGACGACAAGCTTAACTGGAAA ACCTTGTATGAGGTC-3'
	Flag-RPA1-FL-R	5'-TCACATCAATGCACTTCTCCTGATGC-3'
Targeting sequences for shRNA	scramble	5'-TTCTCCGAACGTGTCACGA-3'
	shRPA1 1#	5'-GCAATCCAGTGCCCTATAA-3'
	shRPA1 2#	5'-TTGTTAGCAATCTTCAGGG-3'
